# Reduction in the activity of VTA/SNc dopaminergic neurons underlies aging-related decline in novelty seeking

**DOI:** 10.1038/s42003-023-05571-x

**Published:** 2023-12-02

**Authors:** Qiang Shan, Ye Tian, Hang Chen, Xiaoli Lin, Yao Tian

**Affiliations:** 1 Laboratory for Synaptic Plasticity, Shantou University Medical College, 515041 Shantou, Guangdong China; 2 Chern Institute of Mathematics, Nankai University, 300071 Tianjin, China

**Keywords:** Cognitive ageing, Social behaviour

## Abstract

Curiosity, or novelty seeking, is a fundamental mechanism motivating animals to explore and exploit environments to improve survival, and is also positively associated with cognitive, intrapersonal and interpersonal well-being in humans. However, curiosity declines as humans age, and the decline even positively predicts the extent of cognitive decline in Alzheimer’s disease patients. Therefore, determining the underlying mechanism, which is currently unknown, is an urgent task for the present aging society that is growing at an unprecedented rate. This study finds that seeking behaviors for both social and inanimate novelties are compromised in aged mice, suggesting that the aging-related decline in curiosity and novelty-seeking is a biological process. This study further identifies an aging-related reduction in the activity (manifesting as a reduction in spontaneous firing) of dopaminergic neurons in the ventral tegmental area (VTA) and substantia nigra pars compacta (SNc). Finally, this study establishes that this reduction in activity causally underlies the aging-related decline in novelty-seeking behaviors. This study potentially provides an interventional strategy for maintaining high curiosity in the aged population, i.e., compensating for the reduced activity of VTA/SNc dopaminergic neurons, enabling the aged population to cope more smoothly with the present growing aging society, physically, cognitively and socioeconomically.

## Abstract (web summary)

Behavioral tests together with specific neuronal recordings and manipulation suggest that aging-related decline in social and inanimate novelty seeking is caused by a reduction in the spontaneous firing of midbrain VTA/SNc dopaminergic neurons.

## Introduction

Curiosity is a fundamental mechanism motivating animals to explore novel objects, either inanimate or social (i.e., other animals), in the environment in order to discover potential rewards to exploit, or potential dangers to avoid, ultimately improving survival^1, 2^. The curiosity of human beings is specifically a driving force for scientific discovery, ultimately advancing human civilization^3^. On the other hand, cognitively, curiosity (or novelty seeking) can improve explicit memory^4–8^ and motor learning^9^. Intrapersonally, curiosity is positively associated with life satisfaction and well-being^10,11^. Interpersonally, curiosity is associated with better emotional intelligence^12^ and social intimacy^13^, and less social aggression^14^.

Despite the significance of curiosity or novelty-seeking behaviors, their underlying neural mechanism is largely unknown^15^. Human functional imaging  studies have revealed that high curiosity and novel stimuli increase the activity of the dopaminergic neuron-rich midbrain areas, consisting of the ventral tegmental area (VTA) and the substantia nigra pars compacta (SNc)^16–19^, and the dopaminergic projection-innervated hippocampus^20,21^. These findings provide an anatomical basis for the notion that novelty-seeking is rewarding, like food or water^22,23^; or, more precisely, that novelty can be considered as a dimension of salience^15,24^. Both reward and salience are known to be evaluated by midbrain dopaminergic neurons^25,26^. These findings also provide an anatomical basis for the notion that novelty and curiosity improve learning and memory via a functional loop formed between the VTA and hippocampus^27–29^. Interestingly, multiple lines of studies have shown that midbrain dopaminergic signalling also controls creativity^30–34^, which is tightly associated with curiosity and novelty seeking.

All these facts converge to the conclusion that the midbrain dopaminergic system plays an essential role in curiosity and novelty seeking^15^. Indeed, abnormality in curiosity and novelty seeking is found to be associated with numerous neuro-psychiatric disorders that are caused by, or related to, disfunction of dopaminergic signalling. Generally, excessive novelty preference is associated with hyperdopaminergic disorders such as schizophrenia^35^ (but reduced novelty seeking was seen in a schizophrenic negative-symptom mouse model^36^) and drug addiction^37,38^; conversely, compromised novelty seeking (or related increased apathy) is associated with hypodopaminergic disorders such as Parkinson’s disease^39,40^ and depression^41^. Further limited causal circuitry studies have led to a rough consensus that VTA dopaminergic neurons and SNc dopaminergic neurons are required for social novelty seeking^42,43^ and inanimate novelty seeking^44^, respectively, although this dichotomy is not clear-cut^15,45^.

Curiosity, or the intent of novelty seeking, is an intrinsic motivation, manifesting since infancy^46^. However, curiosity, especially intellectual curiosity, declines as humans age^47–50^. Relatedly, aging is also associated with decreased openness to new experiences^51–55^ and novelty sensitivity^56^, and increased apathy^57^.

Human society is aging at an unprecedented rate^58^. The retirement age is increasing, while science and technology are advancing exponentially. To cope with these challenges, the aged population needs to maintain high curiosity (and creativity) to be competent both professionally and domestically, and thereby socioeconomically productive. On the other hand, it seems that maintaining high curiosity at older ages is predictive of better physical well-being^59–63^ and successful cognitive aging^64^. Therefore, investigating the mechanism underlying the aging-related decline in curiosity and novelty seeking, which remains one of the key questions to be addressed in the field^15^, is of socioeconomic, physical and cognitive significance.

Presumably, socioeconomic factors contribute to this decline. For example, the more isolated lives that the aged population lead after retirement from working life, which potentially results in reduced chances of encountering new people and reduced demand to acquire new knowledge, likely contributes to this decline. However, whether this decline is a biological process is not yet known. This study compared aged and young mice, between which age was the major difference, free from potential complications from socioeconomic factors, and found that seeking behaviors for both social and inanimate novelties are compromised in aged mice, suggesting that the aging-related decline in curiosity and novelty-seeking is a biological process. This study further identified an aging-related functional alteration, i.e., a reduction in the activity (manifesting as a reduction in spontaneous firing) of dopaminergic neurons in the VTA and SNc, and established that this reduction in activity causally underlies the aging-related decline in seeking behaviors for both social and inanimate novelties.

## Results

### Seeking behaviors for both social and inanimate novelties are reduced in aged mice

**Fig. 1:**
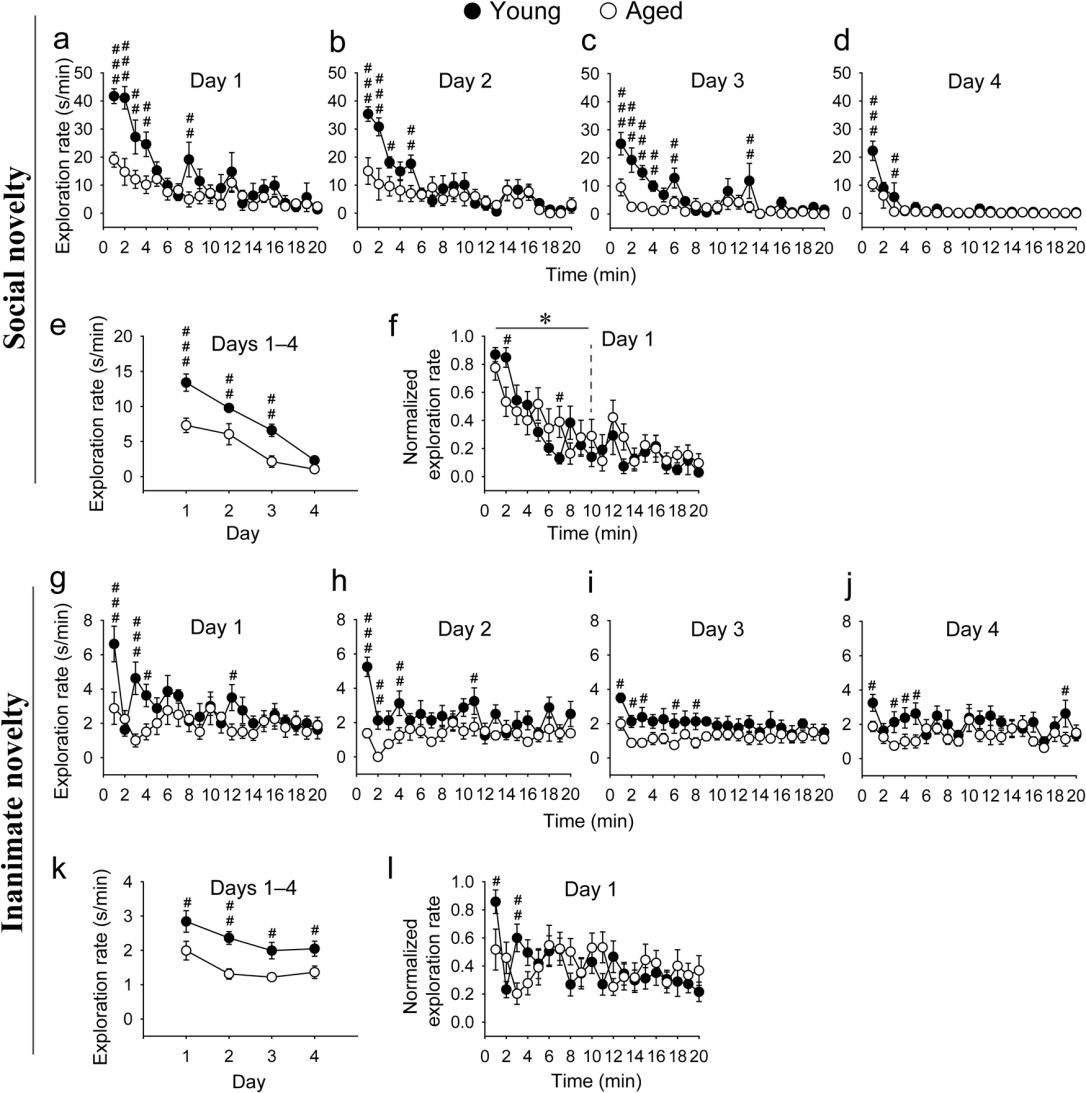
Seeking behaviors for both social and inanimate novelties are reduced in aged mice. **a**–**d** Days 1–4 within-session time courses of the raw exploration rate (i.e., the total exploration time in seconds over each minute of the session duration) for the social novelty. **e** Days 1–4 between-session time course of the raw exploration rate for the social novelty. **f** Day 1 within-session time course of the normalized exploration rate (i.e., for a given mouse, the exploration rate at a given time point divided by the maximum exploration rate (mostly occurring among the first 10 time points) among the 20 time points of the mouse) for the social novelty. **g**–**j** Days 1–4 within-session time courses of the raw exploration rate for the inanimate novelty. **k** Days 1–4 between-session time course of the raw exploration rate for the inanimate novelty. **l** Day 1 within-session time course of the normalized exploration rate for the inanimate novelty. The data displayed in each panel were analyzed by using two-way repeated measures ANOVA, with the statistics displayed as follows: **a** *F*_1,14_ = 15, *P* = 0.002 for age effect; *F*_19,266_ = 12, *P* < 0.001 for time effect; *F*_19,266_ = 3.2, *P* < 0.001 for age × time interaction; **b** *F*_1,14_ = 5.5, *P* = 0.034 for age effect; *F*_19,266_ = 11, *P* < 0.001 for time effect; *F*_19,266_ = 3.2, *P* < 0.001 for age × time interaction; **c** *F*_1,14_ = 14, *P* = 0.002 for age effect; *F*_19,266_ = 9.5, *P* < 0.001 for time effect; *F*_19,266_ = 3.9, *P* < 0.001 for age × time interaction; **d** F_1,14_ = 6.1, *P* = 0.027 for age effect; *F*_19,266_ = 16, *P* < 0.001 for time effect; *F*_19,266_ = 2.2, *P* = 0.003 for age × time interaction; **e** F_1,14_ = 17, *P* < 0.001 for age effect; *F*_3,42_ = 53, *P* < 0.001 for time effect; *F*_3,42_ = 3.7, *P* = 0.019 for age × time interaction; **f** *F*_1,14_ = 0.39, *P* = 0.54 for age effect; *F*_19,266_ = 11, *P* < 0.001 for time effect; *F*_19,266_ = 1.5, *P* = 0.10 for age × time interaction when analyzing all 20 time points. *F*_1,14_ = 0.0, *P* = 0.98 for age effect; *F*_9,126_ = 11, *P* < 0.001 for time effect; *F*_9,126_ = 2.3, *P = 0.02 for age × time interaction when analyzing the first 10 time points only. **g** F_1,14_ = 4.1, p = 0.061 for age effect; *F*_19,266_ = 3.4, *P* < 0.001 for time effect; *F*_19,266_ = 2.5, *P* < 0.001 for age × time interaction; **h** *F*_1,14_ = 19, *P* < 0.001 for age effect; *F*_19,266_ = 2.2, *P* = 0.003 for time effect; *F*_19,266_ = 1.9, *P* = 0.013 for age × time interaction; **i** *F*_1,14_ = 8.9, *P* = 0.010 for age effect; *F*_19,266_ = 1.2, *P* = 0.23 for time effect; *F*_19,266_ = 0.72, *P* = 0.80 for age × time interaction; **j** *F*_1,14_ = 5.8, *P* = 0.030 for age effect; *F*_19,266_ = 1.4, *P* = 0.12 for time effect; *F*_19,266_ = 0.93, *P* = 0.54 for age × time interaction; **k** *F*_1,14_ = 12, *P* = 0.004 for age effect; *F*_3,42_ = 11, *P* < 0.001 for time effect; *F*_3,42_ = 0.52, *P* = 0.67 for age × time interaction; **l** *F*_1,14_ = 0.02, *P* = 0.88 for age effect; *F*_19,266_ = 2.3, *P* = 0.002 for time effect; *F*_19,266_ = 2.0, *P* = 0.007 for age × time interaction when analyzing all 20 time points. *F*_1,14_ = 0.33, *P* = 0.58 for age effect; *F*_9,126_ = 2.5, *P* = 0.013 for time effect; *F*_9,126_ = 2.6, *P* = 0.010 for age × time interaction when analyzing the first 10 time points only. For all panels, *P < 0.05; post-hoc test, ^###^*P* < 0.001, ^##^*P* < 0.01, ^#^*P* < 0.05; *n* = 8 each. All data are presented as mean ± SEM.

To compare novelty-seeking behaviors between aged and young mice, a group of aged mice and a group of young mice were utilized. Each mouse of the two groups was presented for 20 min with a novel object (either a social object (Fig. 1a), i.e., another mouse, or an inanimate object (Fig. 1g)) that the mouse had never encountered, and the exploration behaviors were recorded. As expected, young mice demonstrated frequent exploration of the novel objects during the initial 1 or 2 min of the sessions, and the exploration rate quickly dwindled to a relatively stable low level for the remainder of the sessions (with continuous presence of the objects), which represents a habituation (familiarization) process (Fig. 1a, g). This relatively stable low exploration rate during the late phase of the sessions serves as an index of novelty-independent basal exploration behaviors. In contrast, aged mice demonstrated much less frequent novelty-driven exploration than young mice during the initial phase of the sessions, although both groups’ rates of novelty-independent basal exploration were comparable, which were revealed during the late phase of the sessions (Fig. 1a, g).

Notably, specifically in the case of inanimate novelty seeking, young mice abruptly reduced their exploration rate from a high level during the first minute to a low basal level during the second minute, but recovered to a relatively high level during the third minute, which was followed by a normal habituation process afterwards (Fig. 1g). This brief avoidance behavior can be interpreted as brief neophobia after mice quickly learn a novel inanimate object^15, 65^. It has been proposed that an approach–avoidance strategy is employed by animals specifically when encountering inanimate novelties in order to evaluate potential threat^15^, which can explain the mixture of exploration and avoidance behaviors found here. Such a behavior is consistent with the notion that inanimate novelties can be aversive^42^ or threatening^65^, relative to social novelties, which are usually rewarding, appetitive and reinforcing^66,67^. On the other hand, aged mice also showed a relatively lower exploration rate after the first minute, although this process occurred less abruptly (lasting from the second to fifth minute) and less significantly (possibly due to the relatively lower initial exploration rate of the first minute) than that of young mice (Fig. 1g). Nevertheless, these data suggest that aging is related to a reduction in seeking behaviors for both social and inanimate novelties.

It should be noted that the apparent differential time courses between aged and young mice during the initial phase of the inanimate novelty seeking session might be attributable non-specifically to the differential avoidance of the central area of the test chamber, where the inanimate object was placed. However, analysis of the final acclimation session, in which the object was absent, indicated that aged and young mice spent comparable amounts of time per unit time in the central area during the initial phase as well as the late phase of the session (Supplementary Fig. S1). The initial-phase time course of this blank control (Supplementary Fig. S1) appears distinct from that of the inanimate novelty seeking (Fig. 1g), thereby eliminating the possibility of the non-specific effect.

Each mouse in the two groups was further subjected to the same procedure, i.e., being exposed to the same object in a session, on each of the following 3 consecutive days (Fig. 1b–d, h–j), in order to investigate whether any between-session differences exist between aged and young mice (in addition to the within-session differences, described above) after both groups of mice had reduced their exploration rates to similar low basal levels at the end of the day-1 sessions. Interestingly, in young mice, novelty habituation for both social and inanimate objects from day 1 did not sustain fully across to the next day, as exploration during the initial phase of the day-2 sessions recovered spontaneously (Fig. 1b, h), although to an extent less than that during the initial phase of the day-1 sessions (Fig. 1a, g). The novelty-driven exploration then further habituated to the basal level, similar to that of the day-1 sessions. Similar behaviors (i.e., spontaneous recovery and the following habituation) were also found in the day-3 and day-4 sessions, although spontaneous recovery became less and less significant over the 3 days (i.e., days 2–4) (Fig. 1b–d, h–j), suggesting that novelty habituation is partially retained across sessions, which we term a between-session habituation (familiarization) process. Indeed, when considering the average exploration rate of a session rather than the minute-by-minute exploration rates over the course of the session, apparent between-session novelty-seeking and novelty-habituation processes were revealed across the 4 sessions (i.e., day-1–day-4 sessions) (Fig. 1e, k). Interestingly, both the within-session and between-session time course patterns of the novelty-seeking and novelty-habituation processes in young mice seem to mirror another more-widely studied behavior, classical Pavlovian conditioning^68,69^, of which the conditioned response (CR) is equivalent to the novelty-seeking behaviors here, and of which the conditioning-extinction process is equivalent to the novelty-habituation process here.

Regarding aged mice, the initial within-session exploration rates of the day-2–day-4 sessions (Fig. 1b–d, h–j) (in addition to that of the day-1 session described above, Fig. 1a, g), and the initial between-session exploration rates of the day-1–day-4 sessions (Fig. 1e, k), are lower than those of young mice. These differences disappeared during the late phases of the sessions (or late sessions) except for the between-session exploration rates of inanimate objects (Fig. 1k). It should be noted that the initial within-session exploration rates of the day-2–day-4 sessions of aged mice, relative to young mice (Fig. 1b–d, h–j), should reflect aging-related effects compounded at least by 1-day object memory retention and 1-day novelty habituation retention (both negatively affecting spontaneous novelty recovery) in addition to novelty seeking per se. Both 1-day object memory retention and 1-day novelty habituation retention are likely to be impaired with aging because relevant evidence has shown that long-term memory retention^70^ (which corresponds to the 1-day object memory retention here) and memory extinction retention^71^ (which corresponds to the 1-day novelty habituation retention here) are both impaired with aging. The potential aging-related impairment in either 1-day object memory retention or 1-day novelty habituation retention (of a session), by either of which the object becomes novel again, would likely increase the initial within-session exploration rates during the session on the next day in aged mice, relative to young mice. As such, the determination of novelty seeking of aged mice relative to young mice should mainly rely on the data from the day-1 sessions. However, on the other hand, it seems that the ratios of the initial within-session exploration rate of aged mice to young mice of the day-2 sessions (presumably reflecting an aging-related effect on 1-day object memory retention and 1-day novelty habituation retention in addition to novelty seeking per se) (Fig. 1b, h) are comparable to those of the day-1 sessions (presumably reflecting an aging-related effect on novelty seeking only) (Fig. 1a, g), suggesting the potential impairment in both 1-day object memory retention and 1-day novelty habituation retention in aged mice might be negligible (possibly due to ceiling effects that are caused by the long 20-min exposure of aged mice to the objects) or play a minor role.

Nevertheless, the data presented so far indicate that seeking behaviors for both social and inanimate novelties are reduced in aged mice. Although habituation training was also executed, the data do not directly tell whether novelty habituation is also affected by aging, simply due to the differential initial exploration rates of aged and young mice in each session. To rectify this, for each mouse, the exploration rate at each time point of the day-1 session was normalized against the maximum rate (mostly occurring among the first 10 time points) among the 20 time points of the mouse over the session, and the averaged normalized rates across all aged, and all young, mice for both social and inanimate objects were subsequently plotted against the time points (Fig. 1f, l). It appears that aged mice reduced their normalized exploration rates for social objects slightly slower than young mice (Fig. 1f), suggesting that social novelty habituation is possibly impaired with aging. Note that the statistical significance (of the interaction between normalized exploration rate and time point in the two-way repeated measures ANOVA of the data) for the social novelty experiment was only revealed when the first 10 time points, rather than all 20 time points, were analyzed (Fig. 1f), suggesting that the aging-related impairment of social novelty habituation is moderate if there is any. On the other hand, it is difficult to determine whether this is the case for inanimate novelty habituation because it is confounded with an initial brief abrupt reduction in normalized exploration rates, although it also appears that aged mice reduced their normalized exploration rates for inanimate objects slower than young mice (statistical significance of the interaction between normalized exploration rate and time point in the two-way repeated measures ANOVA of the data was revealed both when the first 10 time points and when all 20 time points were analyzed) (Fig. 1l).

It is also noticeable that social novelty-driven exploration rates (manifesting in the early phase of each session) are generally much higher than the corresponding inanimate novelty-driven exploration rates, which is especially the case during the early phase of the day-1 sessions (6–7-fold difference between the first-minute exploration rates of social and inanimate objects, for both aged and young mice) (Fig. 1a, g). This is consistent with the notion that social novelties are rewarding, appetitive and reinforcing, relative to inanimate novelties^66,67^.

### Spontaneous firing of VTA and SNc dopaminergic neurons is reduced in aged mice

**Fig. 2:**
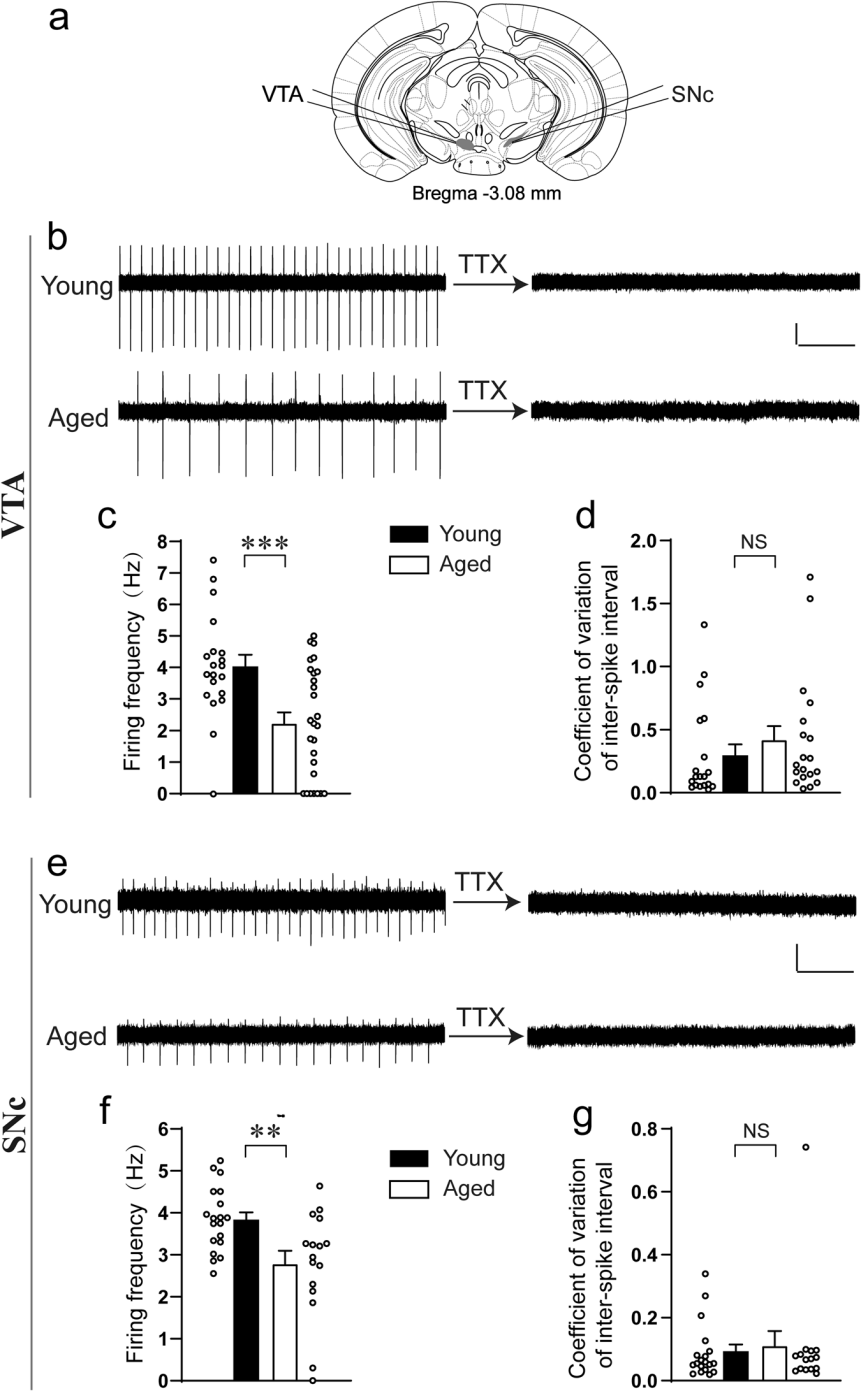
Spontaneous firing of VTA and SNc dopaminergic neurons is reduced in aged mice. **a** Recording locations of the VTA and SNc. **b**, **e** Example spontaneous firing current traces before and after TTX treatment. **c**, **f** Firing frequency (**c** *t* test, *t*_45_ = 3.5, ***P < 0.001, Cohen’s *d* = 1.0, *n* = 20, 27. The frequency of 1 of the 20 cells from young mice and 7 of the 27 cells from aged mice is 0. **f** *t* test, *t*_33_ = 3.0, **P = 0.005, Cohen’s *d* = 1.0, *n* = 19, 16. The frequency of 0 of the 19 cells from young mice and 1 of the 16 cells from aged mice is 0. **d**, **g** Coefficient of variation of inter-spike interval (**d** *t* test, *t*_36_ = 0.86, *P* = 0.39, Cohen’s *d* = 0.28, *n* = 19 each. **g** *t* test, *t*_32_ = 0.35, *P* = 0.73, Cohen’s *d* = 0.12, *n* = 19, 15. NS no significance). Scale bars represent 20 pA (vertical) and 1 s (horizontal). All data are presented as mean ± SEM.

Since VTA/SNc dopaminergic neurons play an essential role in novelty-seeking behaviors, as suggested by previous studies, and novelty-seeking behaviors are reduced in aged mice, as presented above in this study, the question naturally arose whether the activity of VTA/SNc dopaminergic neurons changes in aged mice. Dopaminergic neurons (of healthy young animals) are autonomous pacemakers, firing action potentials in the absence of excitatory synaptic input^72–74^. The electrophysiological parameters of both VTA and SNc dopaminergic neurons were subsequently recorded in both aged and young mice (Fig. 2a). Dopaminergic neurons were identified by the presence of red-fluorescence in DAT-Cre::Ai14 mice. Consistent with literature^74^, dopaminergic neurons in the VTA and SNc of young mice exhibited spontaneous firing with relatively regular rhythms, which can be fully blocked by tetrodotoxin, confirming the nature of the firing as Na^+^ channel-mediated action potentials (Fig. 2b, e). In contrast, the dopaminergic neurons in aged mice fired with a significantly lower frequency (Fig. 2b, c, e, f). Indeed, 7 of the 27 recorded VTA dopaminergic neurons from aged mice failed to fire any action potential, in contrast to only 1 of the 20 VTA dopaminergic neurons from young mice (Fig. 2c). The corresponding numbers for SNc dopaminergic neurons are 1 of 16 versus 0 of 19 (Fig. 2f). Furthermore, the firing rhythm of aged mice also looks slightly irregular compared to that of young mice, and analyzing the coefficient of variation of inter-spike interval indicates this number in aged mice is larger than that of young mice, although the difference does not reach statistical significance (Fig. 2d, g).

Taken together, these data seem to indicate that the basal activity of dopaminergic neurons from both the VTA and SNc is reduced in aged mice. Because of the nature of their preparation, the ex vivo brain slice recordings used in this study are obviously unable to dynamically associate the activity of VTA/SNc dopaminergic neurons with the novelty-seeking behaviors in aged mice that are described above. In order to establish a dynamic association, another method is required, such as cell-type-specific in vivo recordings that are conducted simultaneously with the novelty-seeking behaviors in aged mice. However, this method is possibly too invasive for aged mice to survive. Instead, we next aimed to establish a causal relationship between the reduction in the basal activity of VTA/SNc dopaminergic neurons and the aging-related reduction in novelty-seeking behaviors.

### Chemogenetically suppressing the activity of VTA/SNc dopaminergic neurons in young mice reduces novelty-seeking behaviors, mimicking the behavioral effect found in aged naive mice

**Fig. 3:**
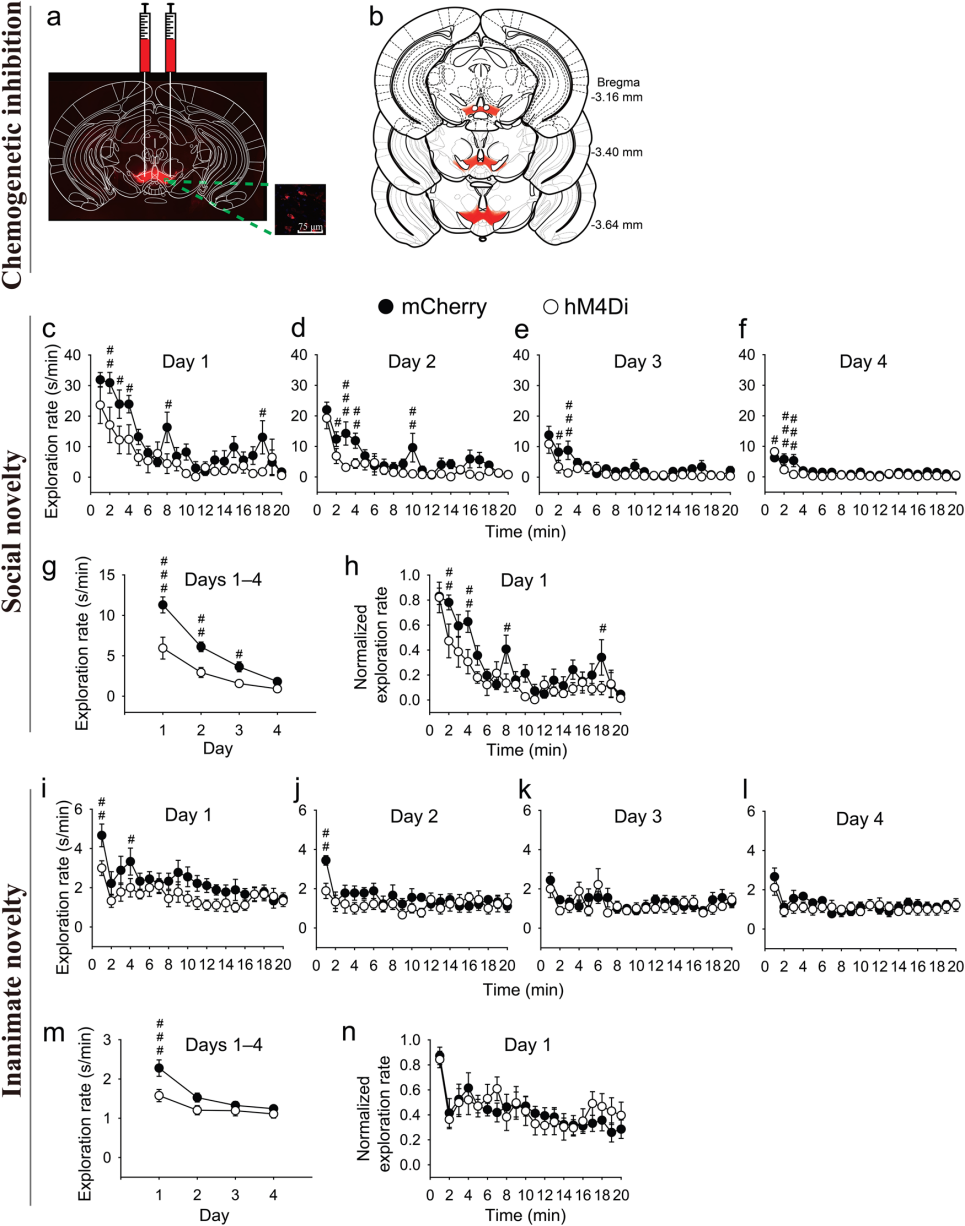
Chemogenetically suppressing the activity of VTA/SNc dopaminergic neurons in young mice reduces novelty-seeking behaviors. **a**, **b** AAV injection location. **c**–**f** Days 1–4 within-session time courses of the raw exploration rate (i.e., the total exploration time in seconds over each minute of the session duration) for the social novelty. **g** Days 1–4 between-session time course of the raw exploration rate for the social novelty. **h** Day 1 within-session time course of the normalized exploration rate (i.e., for a given mouse, the exploration rate at a given time point divided by the maximum exploration rate (mostly occurring among the first 10 time points) among the 20 time points of the mouse) for the social novelty. **i**–**l** Days 1–4 within-session time courses of the raw exploration rate for the inanimate novelty. **m** Days 1–4 between-session time course of the raw exploration rate for the inanimate novelty. **n** Day 1 within-session time course of the normalized exploration rate for the inanimate novelty. The data displayed in each panel of (**c**–**n**) were analyzed by using two-way repeated measures ANOVA, with the statistics displayed as follows: **c** *F*_1,16_ = 10, *P* = 0.006 for chemogenetic effect; *F*_19,304_ = 12, *P* < 0.001 for time effect; *F*_19,304_ = 1.3, *P* = 0.20 for chemogenetic × time interaction; **d** *F*_1,16_ = 14, *P* = 0.002 for chemogenetic effect; *F*_19,304_ = 12, *P* < 0.001 for time effect; *F*_19,304_ = 1.5, *P* = 0.09 for chemogenetic × time interaction; **e** *F*_1,16_ = 9.9, *P* = 0.006 for chemogenetic effect; *F*_19,304_ = 9.1, *P* < 0.001 for time effect; *F*_19,304_ = 1.2, *P* = 0.28 for chemogenetic × time interaction; **f** *F*_1,16_ = 17, *P* < 0.001 for chemogenetic effect; *F*_19,304_ = 12, *P* < 0.001 for time effect; *F*_19,304_ = 1.8, *P* = 0.018 for chemogenetic × time interaction; **g** *F*_1,16_ = 14, *P* = 0.002 for chemogenetic effect; *F*_3,48_ = 70, *P* < 0.001 for time effect; *F*_3,48_ = 6.3, *P* = 0.001 for chemogenetic × time interaction; **h** *F*_1,16_ = 22, *P* < 0.001 for chemogenetic effect; *F*_19,304_ = 15, *P* < 0.001 for time effect; *F*_19,304_ = 1.2, *P* = 0.25 for chemogenetic × time interaction when analyzing all 20 time points. *F*_1,16_ = 18, *P* < 0.001 for chemogenetic effect; *F*_9,144_ = 16, *P* < 0.001 for time effect; *F*_9,144_ = 1.3, *P* = 0.22 for chemogenetic × time interaction when analyzing the first 10 time points only; **i** *F*_1,16_ = 7.2, *P* = 0.016 for chemogenetic effect; *F*_19,304_ = 4.4, *P* < 0.001 for time effect; *F*_19,304_ = 0.79, *P* = 0.72 for chemogenetic × time interaction; **j** *F*_1,16_ = 3.9, *P* = 0.066 for chemogenetic effect; *F*_19,304_ = 2.1, *P* = 0.005 for time effect; *F*_19,304_ = 1.1, *P* = 0.38 for chemogenetic × time interaction; **k** *F*_1,16_ = 0.64, *P* = 0.44 for chemogenetic effect; *F*_19,304_ = 2.1, *P* = 0.005 for time effect; *F*_19,304_ = 0.89, *P* = 0.60 for chemogenetic × time interaction; **l** *F*_1,16_ = 1.3, *P* = 0.28 for chemogenetic effect; *F*_19,304_ = 2.8, *P* < 0.001 for time effect; *F*_19,304_ = 0.50, *P* = 0.96 for chemogenetic × time interaction; **m** *F*_1,16_ = 7.8, *P* = 0.013 for chemogenetic effect; *F*_3,48_ = 17, *P* < 0.001 for time effect; *F*_3,48_ = 2.6, *P* = 0.064 for chemogenetic × time interaction; **n** *F*_1,16_ = 0.16, *P* = 0.69 for chemogenetic effect; *F*_19,304_ = 3.9, *P* < 0.001 for time effect; *F*_19,304_ = 0.52, *P* = 0.95 for chemogenetic × time interaction when analyzing all 20 time points. *F*_1,16_ = 0.0, *P* = 0.95 for chemogenetic effect; *F*_9,144_ = 3.7, *P* < 0.001 for time effect; *F*_9,144_ = 0.40, *P* = 0.94 for chemogenetic × time interaction when analyzing the first 10 time points only. For all panels, post-hoc test, ^###^*P* < 0.001, ^##^*P* < 0.01, ^#^*P* < 0.05; *n* = 9 each. All data are presented as mean ± SEM.

To determine whether this reduction in the activity of VTA/SNc dopaminergic neurons underlies the aging-related reduction in novelty-seeking behaviors, the activity of VTA/SNc dopaminergic neurons in young mice was transiently suppressed by employing an inhibitory chemogenetic hM4Di-DREADD (designer receptor exclusively activated by designer drugs) system^75–77^, which was introduced by injecting hSyn-DIO-hM4Di-mCherry adeno-associated virus (AAV) into the VTA/SNc of young DAT-Cre mice (Fig. 3a, b). An hSyn-DIO-mCherry AAV was also similarly introduced to another group of mice to serve as a control.

**Fig. 4:**
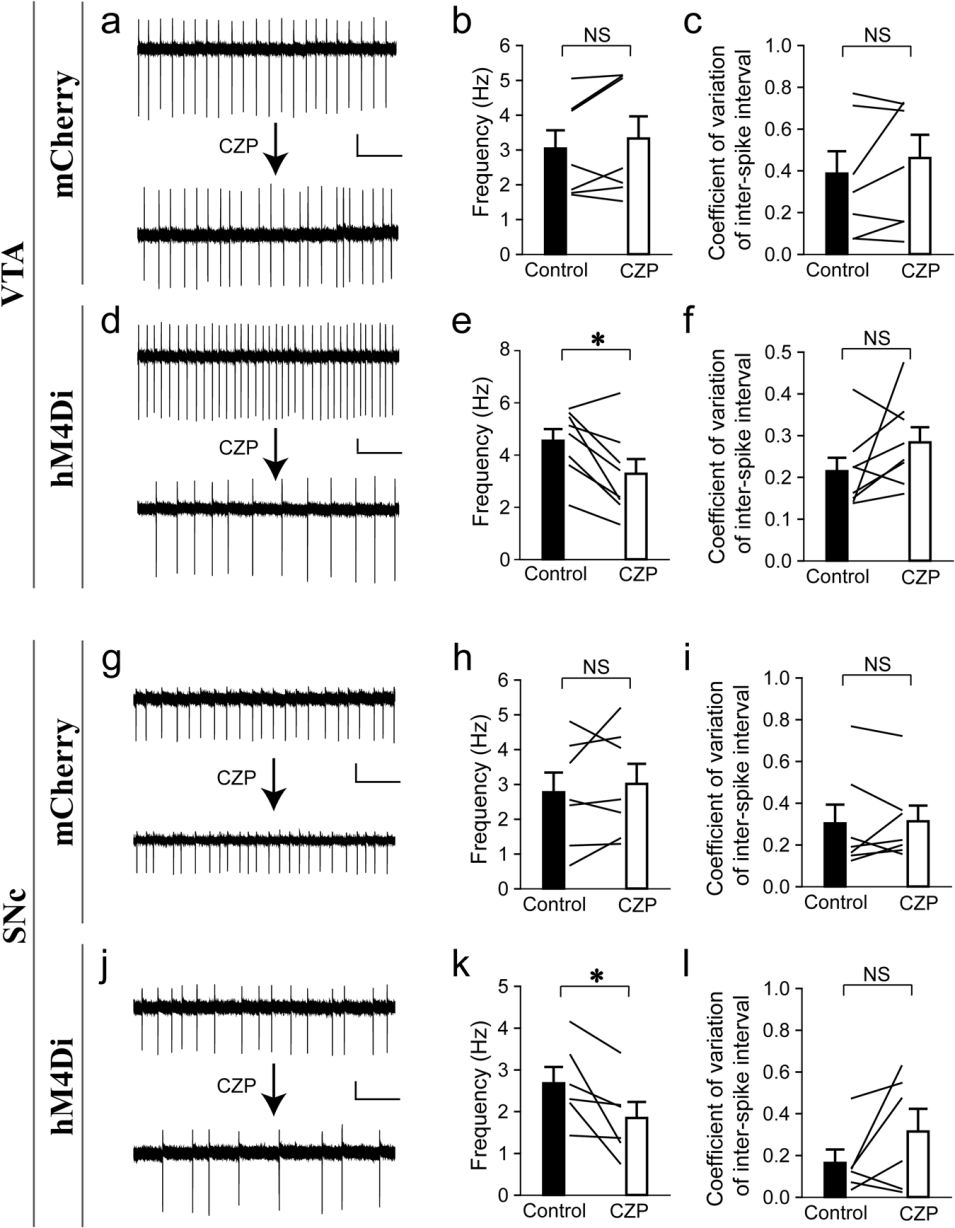
Chemogenetic hM4Di-CZP manipulation reduces spontaneous firing of VTA and SNc dopaminergic neurons in young mice. **a**, **d**, **g**, **j** Example spontaneous firing current traces before and after CZP treatment in indicated conditions. **b**, **e**, **h**, **k** Firing frequency (**b** Paired *t* test, *t*_6_ = 1.4, *P* = 0.22, Cohen’s *d* = 0.52, *n* = 7. **e** Paired *t* test, *t*_7_ = 3.3, *P = 0.013, Cohen’s *d* = 1.2, *n* = 8. **h** Paired *t* test, *t*_6_ = 0.85, *P* = 0.43, Cohen’s *d* = 0.32, *n* = 7. **k** Paired *t* test, *t*_5_ = 2.6, *P = 0.05, Cohen’s *d* = 1.1, *n* = 6). **c**, **f**, **i**, **l** Coefficient of variation of inter-spike interval (**c** Paired *t* test, *t*_6_ = 1.1, *P* = 0.30, Cohen’s *d* = 0.43, *n* = 7. **f** Paired *t* test, *t*_7_ = 1.6, *P* = 0.15, Cohen’s *d* = 0.57, *n* = 8. **i** Paired *t* test, *t*_6_ = 0.26, *P* = 0.80, Cohen’s *d* = 0.10, *n* = 7. **l** Paired *t* test, *t*_5_ = 1.7, *P* = 0.16, Cohen’s *d* = 0.68, *n* = 6). Scale bars represent 40 pA (vertical) and 1 s (horizontal). NS no significance. All data are presented as mean ± SEM.

The efficacy and specificity of this inhibitory DREADD system on the spontaneous firing of VTA/SNc dopaminergic neurons were verified by brain slice recordings. As demonstrated in Fig. 4, the application of the hM4Di agonist clozapine (CZP)^78^ (in order to suppress neuronal activity) significantly reduced the frequency of spontaneous firing of VTA/SNc dopaminergic neurons expressing the hM4Di-mCherry (Fig. 4d, e, j, k), but not those expressing the control mCherry (Fig. 4a, b, g, h). On the other hand, CZP did not seem to significantly affect the firing regularity of VTA/SNc dopaminergic neurons expressing the hM4Di-mCherry (Fig. 4d, f, j, l) or mCherry (Fig. 4a, c, g, i). The hM4Di-CZP effects on the spontaneous firing of VTA/SNc dopaminergic neurons of young DAT-Cre mice here (Fig. 4) seem to mirror the aging effects on the spontaneous firing of VTA/SNc dopaminergic neurons of aged naive mice (Fig. 2) described above.

In terms of behaviors, young DAT-Cre mice expressing either the hM4Di-mCherry or mCherry in VTA/SNc dopaminergic neurons were subjected to the same novelty-seeking behavior tests as those performed on aged and young naive mice (Fig. 1) described above. Analysis of the results revealed that young hM4Di-mCherry-expressing mice that were treated with CZP, compared to young mCherry-expressing mice that were treated with CZP, demonstrated lower within-session and between-session novelty-driven exploration rates (manifesting in the early phase of each session or the early sessions, respectively) for both social and inanimate objects (Fig. 3c–g, i–m), mimicking the behavioral effect found in aged naive mice compared to young naive mice described above (Fig. 1a–e, g–k). Notably, the overall extent of the difference in novelty-driven exploration rates between hM4Di-mCherry- and mCherry-expressing mice in most tests (Fig. 3c–g, i–m) seems smaller than that between aged and young naive mice (Fig. 1a–e, g–k). Furthermore, analysis of the blank control for the inanimate novelty seeking (i.e., the final acclimation session) indicated that hM4Di-mCherry- and mCherry-expressing mice spent comparable amounts of time per unit time in the central area of the test chamber during the initial phase as well as the late phase of the session (Supplementary Fig. S2), eliminating the possibility of the non-specific effect due to differential avoidance of the central area. Collectively, these results suggest that reduction in the activity (manifesting in the reduction of spontaneous firing) of VTA/SNc dopaminergic neurons likely contributes, at least partially, to the aging-related reduction in seeking behaviors for both social and inanimate novelties.

On the other hand, plotting the normalized exploration rates from the day-1 sessions against time points to investigate novelty habituation behaviors indicated that young hM4Di-mCherry-expressing mice that were treated with CZP, relative to young mCherry-expressing mice that were treated with CZP, showed either an expedited reduction of the normalized exploration rates for social novelties (Fig. 3h), or no difference in the reduction of the normalized exploration rates for inanimate novelties (Fig. 3n). Neither result is consistent with the corresponding result of the habituation of social novelty in the case of aged naive mice relative to young naive mice (Fig. 1f) described above, suggesting that reduction in the activity of VTA/SNc dopaminergic neurons does not likely contribute to the aging-related impairment in the habituation of social novelty (if there is any). This conclusion is consistent with a recent study suggesting that another midbrain region, the interpenduncular nucleus, but not the VTA, controls social novelty familiarization (habituation)^66^.

### Chemogenetically enhancing the activity of VTA/SNc dopaminergic neurons in aged mice restores novelty-seeking behaviors, mimicking the behavioral effect found in young naive mice

**Fig. 5:**
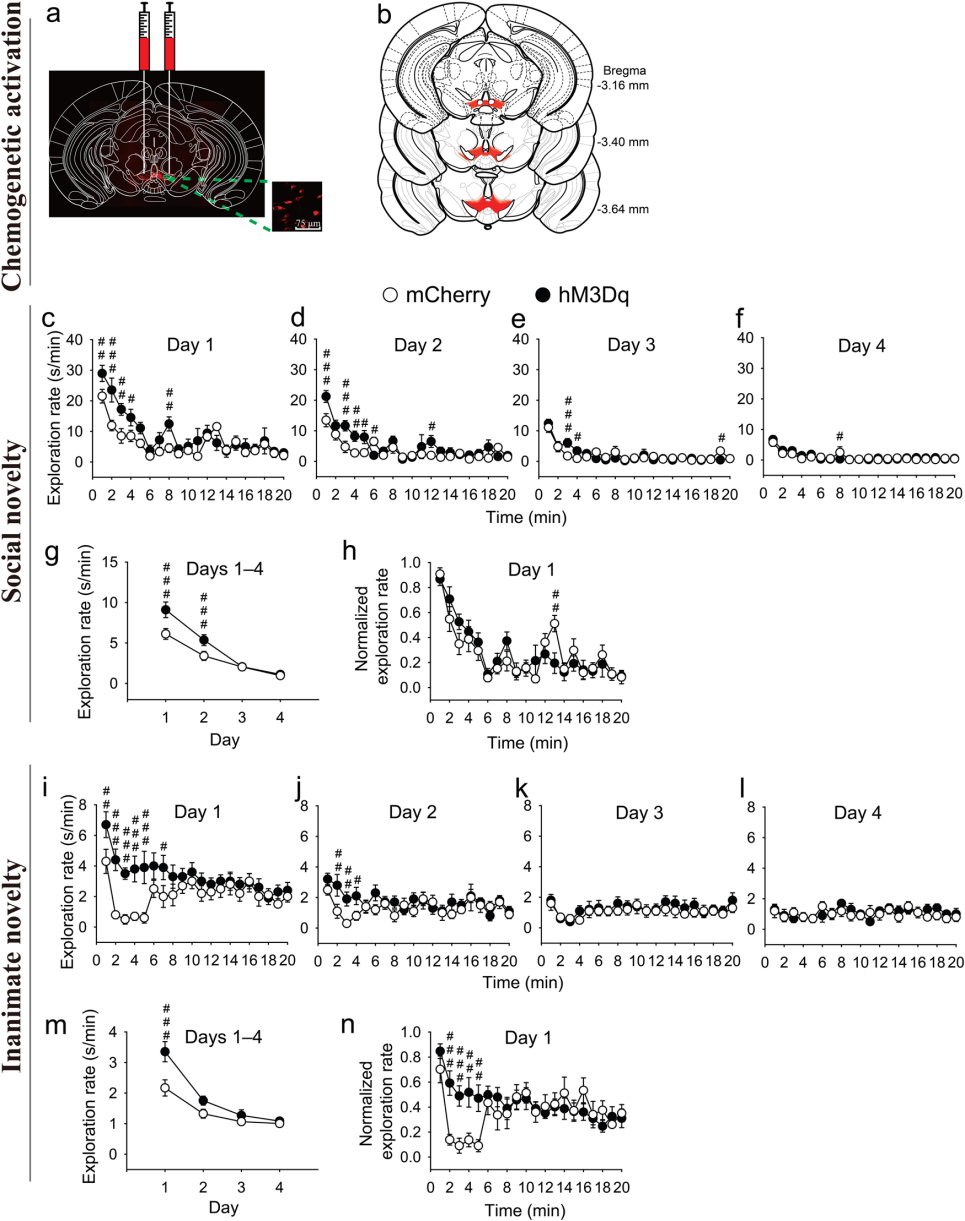
Chemogenetically enhancing the activity of VTA/SNc dopaminergic neurons in aged mice restores novelty-seeking behaviors. **a**, **b** AAV injection location. **c**–**f** Days 1–4 within-session time courses of the raw exploration rate (i.e., the total exploration time in seconds over each minute of the session duration) for the social novelty. **g** Days 1–4 between-session time course of the raw exploration rate for the social novelty. **h** Day 1 within-session time course of the normalized exploration rate (i.e., for a given mouse, the exploration rate at a given time point divided by the maximum exploration rate (mostly occurring among the first 10 time points) among the 20 time points of the mouse) for the social novelty. **i**–**l** Days 1–4 within-session time courses of the raw exploration rate for the inanimate novelty. **m** Days 1–4 between-session time course of the raw exploration rate for the inanimate novelty. **n** Day 1 within-session time course of the normalized exploration rate for the inanimate novelty. The data displayed in each panel of (**c**–**n**) were analyzed by using two-way repeated measures ANOVA, with the statistics displayed as follows: **c** *F*_1,15_ = 17, *P* = 0.001 for chemogenetic effect; *F*_19,285_ = 16, *P* < 0.001 for time effect; *F*_19,285_ = 2.0, *P* = 0.009 for chemogenetic × time interaction; **d** *F*_1,15_ = 28, *P* < 0.001 for chemogenetic effect; *F*_19,285_ = 14, *P* < 0.001 for time effect; *F*_19,285_ = 2.3, *P* = 0.002 for chemogenetic × time interaction; **e** *F*_1,15_ = 0.0, *P* = 0.93 for chemogenetic effect; *F*_19,285_ = 17, *P* < 0.001 for time effect; *F*_19,285_ = 1.6, *P* = 0.051 for chemogenetic × time interaction; **f** *F*_1,15_ = 0.49, *P* = 0.49 for chemogenetic effect; *F*_19,285_ = 11, *P* < 0.001 for time effect; *F*_19,285_ = 0.79, *P* = 0.71 for chemogenetic × time interaction; **g** *F*_1,15_ = 14, *P* = 0.002 for chemogenetic effect; *F*_3,45_ = 298, *P* < 0.001 for time effect; *F*_3,45_ = 18, *P* < 0.001 for chemogenetic × time interaction; **h** *F*_1,15_ = 0.18, *P* = 0.68 for chemogenetic effect; *F*_19,285_ = 17, *P* < 0.001 for time effect; *F*_19,285_ = 1.3, *P* = 0.15 for chemogenetic × time interaction when analyzing all 20 time points. *F*_1,15_ = 2.9, *P* = 0.11 for chemogenetic effect; *F*_9,135_ = 27, *P* < 0.001 for time effect; *F*_9,135_ = 0.63, *P* = 0.77 for chemogenetic × time interaction when analyzing the first 10 time points only. **i** *F*_1,18_ = 9.1, *P* = 0.007 for chemogenetic effect; *F*_19,342_ = 4.4, *P* < 0.001 for time effect; *F*_19,342_ = 2.7, *P* < 0.001 for chemogenetic × time interaction; **j** *F*_1,18_ = 4.4, *P* = 0.051 for chemogenetic effect; *F*_19,342_ = 2.2, *P* = 0.003 for time effect; *F*_19,342_ = 1.3, *P* = 0.20 for chemogenetic × time interaction; **k** *F*_1,18_ = 0.90, *P* = 0.36 for chemogenetic effect; *F*_19,342_ = 1.5, *P* = 0.081 for time effect; *F*_19,342_ = 0.28, *P* = 1.0 for chemogenetic × time interaction; **l** *F*_1,18_ = 0.36, *P* = 0.55 for chemogenetic effect; *F*_19,342_ = 0.95, *P* = 0.52 for time effect; *F*_19,342_ = 0.59, *P* = 0.92 for chemogenetic × time interaction; **m** *F*_1,18_ = 6.4, *P* = 0.021 for chemogenetic effect; *F*_3,54_ = 51, *P* < 0.001 for time effect; *F*_3,54_ = 5.1, *P* = 0.004 for chemogenetic × time interaction; **n** *F*_1,18_ = 2.2, *P* = 0.16 for chemogenetic effect; *F*_19,342_ = 4.0, *P* < 0.001 for time effect; *F*_19,342_ = 3.0, *P* < 0.001 for chemogenetic × time interaction when analyzing all 20 time points. *F*_1,18_ = 16, *P* < 0.001 for chemogenetic effect; *F*_9,162_ = 5.7, *P* < 0.001 for time effect; *F*_9,162_ = 2.5, *P* = 0.010 for chemogenetic × time interaction when analyzing the first 10 time points only. For all panels, post-hoc test, ^###^*P* < 0.001, ^##^*P* < 0.01, ^#^*P* < 0.05; *n* = 8–10. All data are presented as mean ± SEM.

To determine whether compensating for the reduced activity of VTA/SNc dopaminergic neurons in aged mice is able to restore novelty-seeking behaviors, the activity of VTA/SNc dopaminergic neurons in aged mice was transiently enhanced by employing an excitatory chemogenetic hM3Dq-DREADD^75–77^ system, which was introduced by injecting hSyn-DIO-hM3Dq-mCherry AAV into the VTA/SNc of aged DAT-Cre mice (Fig. 5a, b). An hSyn-DIO-mCherry AAV was also similarly introduced to another group of mice to serve as a control.

**Fig. 6:**
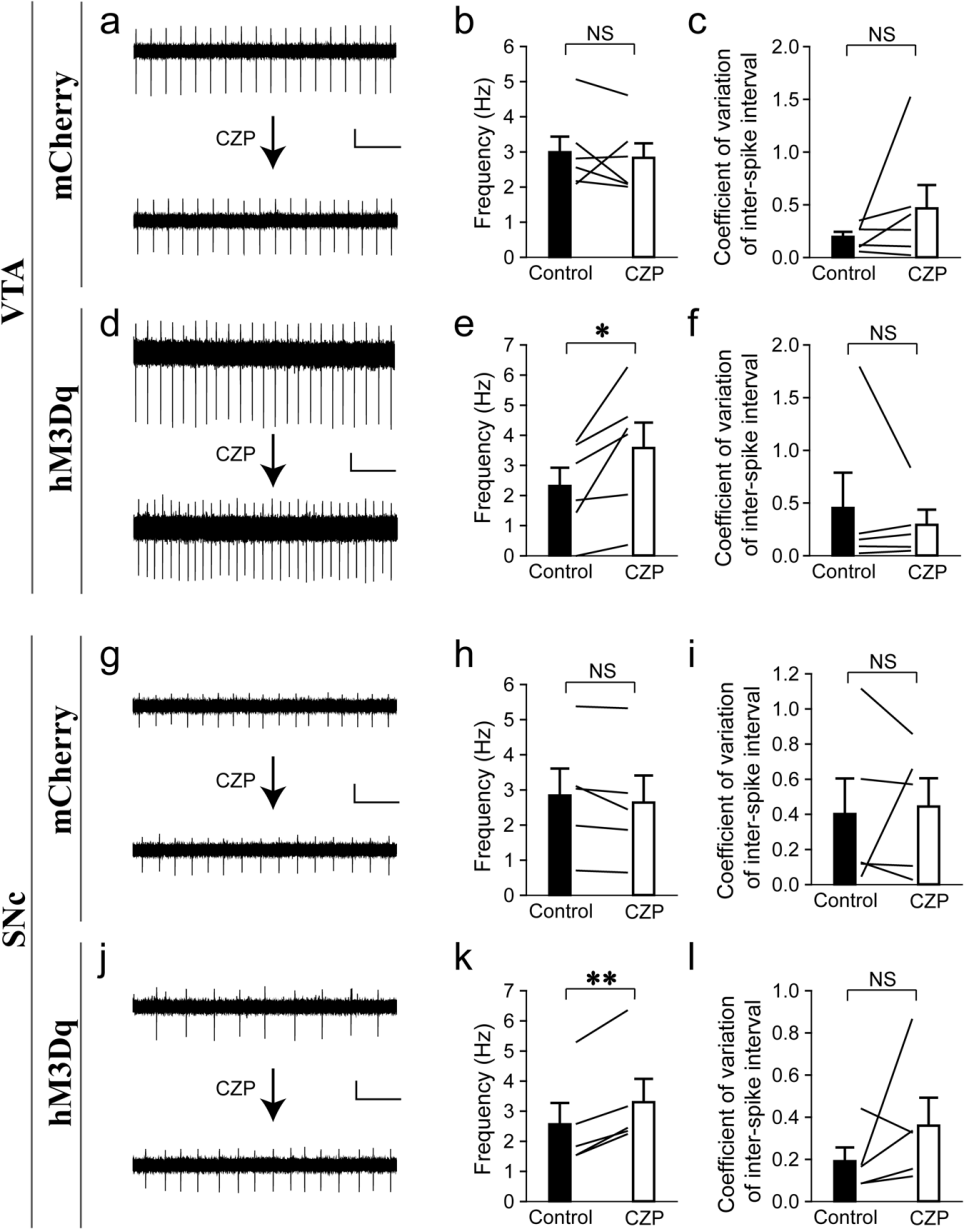
Chemogenetic hM3Dq-CZP manipulation increases spontaneous firing of VTA and SNc dopaminergic neurons in aged mice. **a**, **d**, **g**, **j** Example spontaneous firing current traces before and after CZP treatment in indicated conditions. **b**, **e**, **h**, **k** Firing frequency (**b** Paired *t* test, *t*_5_ = 0.50, *P* = 0.64, Cohen’s *d* = 0.21, *n* = 6. **e** Paired *t* test, *t*_5_ = 2.9, *P = 0.035, Cohen’s *d* = 1.2, *n* = 6. **h** Paired *t* test, *t*_4_ = 1.8, *P* = 0.15, Cohen’s *d* = 0.79, *n* = 5. **k** Paired *t* test, *t*_4_ = 7.4, **P = 0.002, Cohen’s *d* = 3.3, *n* = 5). **c**, **f**, **i**, **l** Coefficient of variation of inter-spike interval (**c** Paired *t* test, *t*_5_ = 1.3, *P* = 0.24, Cohen’s *d* = 0.55, *n* = 6. **f** Paired *t* test, *t*_4_ = 0.81, *P* = 0.46, Cohen’s *d* = 0.36, *n* = 5. **i** Paired *t* test, *t*_4_ = 0.29, *P* = 0.79, Cohen’s *d* = 0.13, *n* = 5. **l** Paired *t* test, *t*_4_ = 1.2, *P* = 0.29, Cohen’s *d* = 0.55, *n* = 5). Scale bars represent 20 pA (vertical) and 1 s (horizontal). NS no significance. All data are presented as mean ± SEM.

The efficacy and specificity of this excitatory DREADD system on the spontaneous firing of VTA/SNc dopaminergic neurons were verified by brain slice recordings. As demonstrated in Fig. 6, the application of the hM3Dq agonist CZP^78^ (in order to enhance neuronal activity) significantly increased the frequency of spontaneous firing of the VTA/SNc dopaminergic neurons expressing the hM3Dq-mCherry (Fig. 6d, e, j, k), but not those expressing the control mCherry (Fig. 6a, b, g, h). On the other hand, CZP did not seem to significantly affect the firing regularity of VTA/SNc dopaminergic neurons expressing the hM3Dq-mCherry (Fig. 6d, f, j, l) or mCherry (Fig. 6a, c, g, i). These data suggest that the hM3Dq-CZP manipulation is able to restore the spontaneous firing of VTA/SNc dopaminergic neurons of aged DAT-Cre mice (Fig. 6), at least partially, to the level of young naive mice (Fig. 2).

In terms of behaviors, aged DAT-Cre mice expressing either the hM3Dq-mCherry or mCherry in the VTA/SNc dopaminergic neurons were subjected to the same novelty-seeking behavior tests as those performed on aged and young naive mice (Fig. 1), and young hM4Di-mCherry- and mCherry-expressing DAT-Cre mice (Fig. 3), described above. Analysis of the results revealed that aged hM3Dq-mCherry-expressing mice that were treated with CZP, compared to aged mCherry-expressing mice that were treated with CZP, demonstrated higher within-session and between-session novelty-driven exploration rates (manifesting in the early phase of each session or early sessions, respectively) for both social and inanimate objects (Fig. 5c–g, i–m), mimicking the behavioral effect found in young naive mice compared to aged naive mice described above (Fig. 1a–e, g–k). Notably, the overall extent of the difference in novelty-driven exploration rates between hM3Dq-mCherry- and mCherry-expressing mice in most tests (Fig. 5c–g, i–m) seems smaller than that between young and aged naive mice (Fig. 1a–e, g–k). Nevertheless, these results suggest that compensating for the reduced activity (manifesting in the increase of spontaneous firing by enhancing the activity) of VTA/SNc dopaminergic neurons in aged mice is able to restore, at least partially, seeking behaviors for both social and inanimate novelties. This conclusion mirrors the corresponding conclusion drawn in the case of young hM4Di-mCherry- compared to mCherry-expressing DAT-Cre mice, described above (Fig. 3).

Notably, specifically for the day-1 sessions (and possibly the day-2 sessions as well) in the case of the inanimate novelty, it seems that the abrupt temporary reduction in the exploration rate immediately after the first minute disappeared completely in the hM3Dq-mCherry-expressing mice that were treated with CZP, relative to the control mCherry-expressing mice that were treated with CZP, which demonstrated the abrupt temporary reduction in the exploration rate from the second to fourth minutes (Fig. 5i). Note that the abrupt temporary reduction in the exploration rate still exists in young naive mice, despite being as brief as 1 min (i.e., the second minute only) (Fig. 1g). As described above, this abrupt temporary reduction in the exploration rate can be interpreted as brief neophobia after mice quickly learn a novel inanimate object^15,65^. This neophobia likely reflects an anxiety-like state. Indeed, the time spent exploring a novel inanimate object is sometimes used to measure the state of anxiety (the less time, the higher the state of anxiety) (e.g., Anthony et al. ^79^). A previous study suggests that VTA dopaminergic projections to the basal lateral amygdala negatively regulate the state of anxiety^80^. Therefore, chemogenetic enhancement of the activity of VTA dopaminergic neurons should alleviate the anxiety-state-associated neophobia, and thereby lessen the abrupt temporary reduction in the exploration rate. Notably, previous studies also suggest that the activity of dopaminergic neurons in the lateral, but not medial, SNc is positively associated with aversion^65,81^, and thereby potential activation of the lateral SNc would increase aversion to the novel object. However, this effect does not likely dominate in the case reported here, as chemogenetic manipulation was restricted to the medial, but not the lateral, SNc (Fig. 5a, b).

Furthermore, analysis of the blank control for the inanimate novelty seeking (i.e., the final acclimation session) indicated that hM3Dq-mCherry- and mCherry-expressing mice spent comparable amounts of time per unit time during the initial phase of the session in the central area of the test chamber, where the inanimate object was yet to be placed during the novelty seeking session (Supplementary Fig. S3), eliminating the possibility of the non-specific effect due to differential avoidance of the central area. Notably, hM3Dq-mCherry-expressing mice gradually increased their amounts of time spent per unit time in the central area over the course of the session, which were significantly higher than those of mCherry-expressing mice during the late phase of the session (Supplementary Fig. S3). This difference can be explained by the proposal raised above that chemogenetic enhancement of the activity of VTA dopaminergic neurons can alleviate anxiety-state-associated behaviors. It is usually thought that the higher the anxiety state of a mouse, the more often the mouse avoids the central area of a field. On the other hand, hM3Dq-mCherry-expressing mice demonstrated  quite distinct time courses of the novelty seeking session and its blank control, i.e., novelty exploration rates gradually declined over the course of the novelty seeking session (Fig. 5i), whereas amounts of time spent in the central area per unit time gradually increased over the course of the blank control session (Supplementary Fig. S3). In contrast, mCherry-expressing mice demonstrated  relatively less distinct corresponding time courses. These differential demonstrations between the two groups of mice suggest that a more drastic novelty seeking effect for hM3Dq-mCherry-expressing mice and a more significant difference in novelty seeking effect between hM3Dq-mCherry- and mCherry-expressing mice would be expected, if the raw novelty exploration data were adjusted against their respective blank control data. In addition, the fact that hM3Dq-mCherry-expressing mice demonstrated high object exploration rates during the early novelty-seeking phase relative to the late habituation phase of the novelty seeking session (Fig. 5i), in contrast to the low amounts of time mice spent in the central area per unit time during the early phase relative to the late phase of the blank control session (Supplementary Fig. S3), imply that novelties are appetitive, so that it can defeat, and even dominate over, the aversive effect of the objects’ locations, which mirrors similar observations on the curiosity of humans^22^.

Regarding habituation behaviors, plotting the normalized exploration rates from the day-1 sessions against time points indicated that aged hM3Dq-mCherry-expressing mice that were treated with CZP, relative to aged mCherry-expressing mice that were treated with CZP, showed no difference in the reduction of the normalized exploration rates for social novelties (Fig. 5h). This result is inconsistent with the corresponding result in the habituation of social novelty in the case of young naive mice relative to aged naive mice (Fig. 1f), suggesting that compensating for the reduced activity of VTA/SNc dopaminergic neurons is not likely able to reverse the aging-related impairment in the habituation of social novelty (if there is any), mirroring the corresponding conclusion drawn in the case of young hM4Di-mCherry- compared to mCherry-expressing DAT-Cre mice, described above (Fig. 3h). In the case of the habituation of inanimate novelty, no conclusion was drawn because of the complication from the differential abrupt temporary reduction of the normalized exploration rates during the early phases of the sessions between aged hM3Dq-mCherry- and mCherry-expressing DAT-Cre mice (Fig. 5n), as discussed above.

## Discussion

This study finds that an aging-related reduction in seeking behaviors for both social and inanimate novelties exists in mouse models, suggesting that it is a biological process. This study also identifies an aging-related reduction in the activity (manifesting as a reduction in spontaneous firing) of VTA and SNc dopaminergic neurons, and finally establishes a causal relationship between these two aging-related changes. However, by comparing the data presented in Figs. 1,  3 and 5, it appears that the extent of the reduction in novelty-seeking behaviors by DREADD-suppressing VTA/SNc dopaminergic neuron activity in young mice (Fig. 3) and the extent of the increase in novelty-seeking behaviors by DREADD-enhancing VTA/SNc dopaminergic neurons in aged mice (Fig. 5) are both smaller than the extent by which aged naive mice reduced novelty-seeking behaviors relative to young naive mice (Fig. 1). This difference is probably attributable to the DREADD manipulation not being as effective as age in affecting the activity of VTA/SNc dopaminergic neurons. This difference could also imply that the reduction in the activity of VTA/SNc dopaminergic neurons only plays a partial role in the aging-related reduction in novelty-seeking behaviors, and that other underlying neural mechanisms need to be elucidated to fully explain this aging-related behavioral deficit.

The data of this study also imply that novelty habituation might also be compromised in aged mice, which is possibly not attributed to the aging-related reduction in the activity of VTA/SNc dopaminergic neurons. Obviously, more appropriately designed experiments are required to verify whether these conclusions are true, and to investigate the relevant underlying neural mechanisms.

It is known that the number of neurons in the SNc decreases with aging. In humans, 6% of medial SNc dopaminergic neurons and 2% of lateral SNc dopaminergic neurons are lost per decade^82^. This neuronal loss is proportional to the decrease in striatal dopamine availability^83^. However, whether aging is associated with any functional changes in dopaminergic neurons, especially in the VTA, is largely unknown. This study reports an aging-related reduction in VTA and SNc dopaminergic neuron activity, manifesting as reduced spontaneous firing (Fig. 2). The reduction in firing rate likely reflects reduced function of excitatory channels such as Na^+^ and Ca^2+^ channels^84,85^ or enhanced function of inhibitory channels such as K^+^ channels^86,87^.

Multiple previous studies based on various species and behavioral paradigms have shown that spontaneous firing of midbrain dopaminergic neurons increases in response to the introduction of a novel stimulus^88–92^, and this increase in firing seems to be required for novelty-seeking behaviors^44^. This increase in firing rapidly declines with the continuous presence of the stimulus. The time course (i.e., a quick rise followed by gradual decline) of this microscopic electrophysiological firing rate mirrors the time course of the macroscopic behavioral novelty-exploration rate reported in this study. Increased firing of dopaminergic neurons would facilitate dopamine release at downstream regions. Consistently, a novel stimulus also increases dopamine levels at the dopamine releasing regions, such as the nucleus accumbens and medial prefrontal cortex^93,94^. Therefore, the aging-related reduction in spontaneous firing of VTA/SNc dopaminergic neurons and the consequent reduction in downstream dopamine release would reduce novelty-seeking behaviors, as demonstrated in this study.

Interestingly, impairment in novelty-seeking or related behaviors, such as decreased curiosity or increased apathy, are frequently seen in Alzheimer’s disease patients^95–97^. Indeed, apathy or impaired novelty processing can even positively predict the extent of cognitive decline in patients with Alzheimer’s disease^98–101^. Conversely, novelty preference is positively associated with better cognitive function in aged adults^64,102,103^ and even a reduced risk of Alzheimer’s disease^104^. Therefore, considering the fact that novelty and curiosity improve learning and memory via the participation of VTA/SNc dopaminergic neurons^27–29^, we propose that the reduction in the activity of these neurons underlying the natural aging-related decline in novelty-seeking behaviors might serve as a predisposing factor for the pathogenesis of Alzheimer’s disease. This proposal provides a biological basis for the epidemiological finding that age is the greatest risk factor for Alzheimer’s disease^105,106^. Interventions to enhance VTA/SNc dopaminergic neuron activity and the consequent downstream dopamine release should provide a possible pathway to preventing, delaying, or ameliorating the cognitive decline in Alzheimer’s disease. Consistent with this proposal, mouse model studies indicate that administration of the dopamine precursor, levodopa, ameliorates learning and memory deficits in Alzheimer’s disease^107,108^.

More widely, interventions to enhance the VTA/SNc dopaminergic neuron activity in the aged population should not only help to maintain novelty-seeking, curiosity and creativity, but also improve related cognitive, interpersonal and intrapersonal well-being, and thereby enable the aged population to cope more smoothly, both biomedically and socioeconomically, with the aging society that is growing at an unprecedented rate^1,47^.

## Methods

### Animals

Male, 2–4 months old (young adults) or 20–24 months old (aged adults), C57BL/6J, DAT-Cre (JAX 006660, B6.SJL-Slc6a3tm1.1(cre)Bkmn/J), and Ai14 (JAX 007914, B6.Cg-Gt(ROSA)26Sortm14(CAG-tdTomato)Hze/J) mice were used in this study. The DAT-Cre and Ai14 transgenes were maintained hemizygously and homozygously, respectively. The DAT-Cre and Ai14 mice were crossed to create DAT-Cre::Ai14 mice, in which the red fluorescent protein, tdTomato, is selectively expressed in the DAT-positive cells, i.e., putatively the dopaminergic neurons.

Genotyping was performed following procedures described previously^109^. The sequences of PCR primers, recommended by the vendor, are: 5´-TGGCTGTTGGTGTAAAGTGG-3´ and 5´-CCAAAAGACGGCAATATGGT-3´ for DAT-Cre (5´-TGGCTGTTGGTGTAAAGTGG-3´ and 5´-GGACAGGGACATGGTTGACT-3´ for their wild-type littermates); 5´-CTGTTCCTGTACGGCATGG-3´ and 5´-GGCATTAAAGCAGCGTATCC-3´ for Ai14 (5´-AAGGGAGCTGCAGTGGAGTA-3´ and 5´-CCGAAAATCTGTGGGAAGTC-3´ for their wild-type littermates).

Mice were maintained in a 12-h light/12-h dark cycle in a temperature (21–25 °C)- and humidity (50–65%)-controlled environment, with food and water ad libitum. All procedures were conducted according to the ethical guidelines approved by the Medical Animal Care & Welfare Committee of Shantou University Medical College. We have complied with all relevant ethical regulations for animal testing.

### Viruses

Adeno-associated viruses (AAVs), AAV9-hSyn-DIO-hM4Di-mCherry, AAV9-hSyn-DIO-hM3Dq-mCherry, and AAV9-hSyn-DIO-mCherry, at concentrations of 2.0–5.0 × 10^12^ vector genomes per ml, were used in this study. In order to confirm the identities of the various AVVs, each batch was subjected to PCR and partial sequencing, following procedures described previously^109^.

### Stereotaxic surgeries

Stereotaxic surgery was performed following standard protocols^109^. Briefly, mice were anesthetized by using pentobarbital sodium dissolved in sterile saline (intraperitoneal injection, 80 mg/kg). The three-dimensional coordinates relative to the Bregma of the VTA/SNc (anterior–posterior −3.4 mm; medial–lateral ± 0.5 mm; dorsal–ventral −4.2 mm) were obtained from the standard brain map of C57BL/6 mice^110^. With these coordinates, most of the VTA and the medial SNc are targeted (note that the medial but not the lateral SNc controls inanimate novelty seeking^44^). Each type of virus was bilaterally injected into the VTA/SNc, 0.5 μl per side, by using glass capillaries following procedures described previously^109^. After surgery, mice were allowed to recover for at least seven days before proceeding to behavioral tests, after which mice were sacrificed for the verification of injection locations by using fluorescent microscopy (methods described below). Only mice with correct injection locations were included in the final analysis.

### Behavior

#### Social novelty-seeking behaviors

This protocol is adapted from  Gunaydin et al. ^43^. Briefly, a test mouse was left alone in its home cage for 5 min, after its cagemates were temporarily removed. A stranger mouse (6–8 weeks old, male, C57BL6/J) that the test mouse had never encountered was subsequently introduced into the test mouse’s home cage for 20 min (each test mouse was paired with a unique stranger mouse). The same procedure with the same test mouse and the same stranger mouse was repeated once a day in the following 3 consecutive days. The two mice were video-recorded during the sessions. The time that the test mouse spent sniffing the stranger mouse’s snout, flank or anogenital area, and grooming or pursuing the stranger mouse as the stranger mouse actively explored the cage, was recorded as the exploration time of the test mouse. The advantage of this novelty-seeking behavior protocol is discussed in Supplementary Discussion.

#### Inanimate novelty-seeking behaviors

This protocol is adapted from Watson et al. ^111^. Briefly, a mouse was acclimatized to a test chamber for 20 min each day for 4 consecutive days. On the 5th day, an inanimate object with a specific geometric shape that the test mouse had never encountered was fixed (by using double-sided tape) onto the floor at the center of the chamber, and the test mouse was introduced to the chamber and allowed to roam for 20 min. The same procedure with the same mouse and the same inanimate object was repeated once a day for the following 3 consecutive days. The mouse was video-recorded during the sessions. The time that the mouse spent sniffing, touching, and directing attention to the object with the nose within 1 cm of the object, but not climbing on or chewing the object, was recorded as the object exploration time. The 4th acclimation session, in which the object was absent, was also video-recorded, and the time that the mouse spent in the central area of the test chamber, where the object was yet to be placed during the novelty seeking sessions, served as the blank control. The advantage of this novelty-seeking behavior protocol is discussed in Supplementary Discussion.

#### Chemogenetic manipulations on behaviors

The chemogenetic manipulations were achieved by injecting mice with CZP^78^ (intraperitoneal injection, 0.01 mg/kg, achieved by administering 0.5 μg/ml stock solution in sterile saline at 20 μl/g, Sigma) 40 min prior to each behavior session. The chemogenetic manipulations were executed at least 10 days after virus injection to allow enough time for virus infection and expression.

### Electrophysiology

Aged or young DAT-Cre::Ai14 mice, young DAT-Cre mice injected with AAV9-hSyn-DIO-hM4Di-mCherry or AAV9-hSyn-DIO-mCherry into the VTA/SNc, or aged DAT-Cre mice injected with AAV9-hSyn-DIO-hM3Dq-mCherry or AAV9-hSyn-DIO-mCherry into the VTA/SNc, were anesthetized by using pentobarbital sodium (intraperitoneal injection, 100 mg/kg, Sigma) and decapitated. The brain was quickly removed and coronal brain slices (270-μm thickness) containing the VTA/SNc were sectioned on a vibratome (Leica VT1200S) stage in an ice-cold dissection solution containing (in mM): 2.5 KCl, 0.5 CaCl_2_, 7 MgCl_2_, 1.2 NaH_2_PO_4_, 26 NaHCO_3_, 10 glucose and 200 sucrose (saturated with 95% O_2_/5% CO_2_; pH 7.4; osmolarity 295–305 mOsm; Sigma). Slices were allowed to recover at room temperature for at least 1 h in a submerged incubation chamber containing 1 mM kynurenic acid (Sigma) in artificial cerebrospinal fluid (ACSF) solution containing (in mM): 126 NaCl, 2.5 KCl, 2.4 CaCl_2_, 1.2 MgCl_2_, 1.2 NaH_2_PO_4_, 26 NaHCO_3_, and 10 glucose (saturated with 95% O_2_/5% CO_2_; pH 7.4; osmolarity 310–320 mOsm; Sigma). Slices were subsequently transferred from the incubation chamber to the recording chamber, where slices were continuously perfused in oxygenated (95% O_2_/5% CO_2_) ACSF solution (1–2 ml/min) at 31–32 °C. Cells were visualized under a 40× water-immersion objective of an upright microscope (Nikon FN1) with the aid of an infrared differential interference contrast (IR-DIC) optic and an infrared camera (DAGE-MTI IR-1000E). The VTA/SNc dopaminergic neurons were identified by the presence of tdTomato or mCherry red fluorescence.

To detect spontaneous firing, cell-attached recording in voltage-clamp mode (holding voltage for electrode is 0 mV) was conducted using a Multiclamp 700B amplifier equipped with pClamp10.6 software (Molecular Devices). Signals were filtered at 10 kHz and digitized at 50 kHz with a Digidata 1550B digitizer (Molecular Devices). Recording pipettes were filled with a solution containing (in mM): 120 CsMeSO_3_, 15 CsCl, 4 NaCl, 2 MgCl_2_, 10 HEPES, 0.4 EGTA, 3 QX-314, 2 Na_2_ATP and 0.33 Na_3_GTP (pH 7.3 and osmolarity 280–290 mOsm; Sigma). Recordings were conducted for at least 2 min on each cell. In some cases, cells were further recorded in the presence of 0.5 µM tetrodotoxin (TTX, Tocris) or 0.01 μM CZP.

### Histology

Mice were anesthetized with pentobarbital sodium (intraperitoneal injection, 80 mg/kg, Sigma), and transcardially perfused with cold saline, which was followed by paraformaldehyde (PFA, 4%, Sigma) in cold phosphate buffer (PB containing 0.019 M NaH_2_PO_4_ and 0.081 M Na_2_HPO_4_, pH = 7.4; Sigma). The brains were removed and post-fixed overnight in 4% PFA, and quickly rinsed in PB before sectioning. Coronal brain slices with a 40-μm thickness were serially sectioned on a vibratome stage (Leica VT1000S).

Direct imaging of the native fluorescence of mCherry was employed to determine the locations of virus injections. To achieve this, brain slices from the DAT-Cre mice injected with AAV9-hSyn-DIO-hM3Dq-mCherry, AAV9-hSyn-DIO-hM4Di-mCherry, or AAV9-hSyn-DIO-mCherry viruses, were rinsed in ddH_2_O for 10 min and subsequently mounted onto glass slides in a thin layer of mounting medium (Fluoroshield with DAPI, Sigma), and covered with glass coverslips. The slices were imaged at a rough scale on an upright fluorescent microscope (PerkinElmer, Vectra Slide Analysis System), or at a cellular level on a laser scanning confocal fluorescent microscope (Zeiss LSM 800).

### Statistics and reproducibility

Specifically for the electrophysiology data, spiking events of neurons were acquired and detected using the Clampfit (10.6, Molecular Devices) template search function. The coefficient of variation of inter-spike interval (i.e., the time between two adjacent spikes) of a neuron was only calculated for neurons with more than two spiking events, as the standard deviation divided by the mean of all inter-spike intervals of the neuron.

Results were analyzed by using SigmaPlot 12.5 (Systat) or SPSS 25 (IBM) and expressed as the mean ± SEM. Two-way repeated measures ANOVA followed by Bonferroni post-hoc tests was used for analyzing the effects of age and session time point on novelty seeking behaviors. Two-tailed unpaired or paired *t* test was used for analyzing the effect of age or CZP application on electrophysiology, respectively. A *P* value less than 0.05 was considered statistically significant.

### Supplementary information

Supplementary Information

Description of Supplementary Materials

Supplementary Data

## Data Availability

All data underlying figures are provided in the Supplementary Data file. Other data are available upon reasonable request to the corresponding author.

## References

[1] Düzel E, Bunzeck N, Guitart-Masip M, Düzel S (2010). NOvelty-related motivation of anticipation and exploration by dopamine (NOMAD): implications for healthy aging. Neurosci. Biobehav. Rev..

[2] Loewenstein G (1994). The psychology of curiosity: a review and reinterpretation. Psychol. Bull..

[3] Kidd C, Hayden BY (2015). The psychology and neuroscience of curiosity. Neuron.

[4] Ballarini F, Martínez MC, Díaz Perez M, Moncada D, Viola H (2013). Memory in elementary school children is improved by an unrelated novel experience. PLoS ONE.

[5] Fenker DB (2008). Novel scenes improve recollection and recall of words. J. Cognit. Neurosci..

[6] Schomaker J, van Bronkhorst MLV, Meeter M (2014). Exploring a novel environment improves motivation and promotes recall of words. Front. Psychol..

[7] Schomaker J (2019). Unexplored territory: beneficial effects of novelty on memory. Neurobiol. Learn. Mem..

[8] McGillivray S, Murayama K, Castel AD (2015). Thirst for knowledge: the effects of curiosity and interest on memory in younger and older adults. Psychol. Aging.

[9] Ruitenberg MFL, Koppelmans V, Seidler RD, Schomaker J (2022). Novelty exposure induces stronger sensorimotor representations during a manual adaptation task. Ann. N. Y. Acad. Sci..

[10] Gallagher MW, Lopez SJ (2007). Curiosity and well-being. J. Posit. Psychol..

[11] Kashdan TB, Steger MF (2007). Curiosity and pathways to well-being and meaning in life: traits, states, and everyday behaviors. Motiv. Emot..

[12] Leonard NH, Harvey M (2007). The trait of curiosity as a predictor of emotional intelligence. J. Appl. Soc. Psychol..

[13] Kashdan TB, McKnight PE, Fincham FD, Rose P (2011). When curiosity breeds intimacy: taking advantage of intimacy opportunities and transforming boring conversations. J. Pers..

[14] Kashdan TB (2013). Curiosity protects against interpersonal aggression: cross-sectional, daily process, and behavioral evidence. J. Pers..

[15] Tapper AR, Molas S (2020). Midbrain circuits of novelty processing. Neurobiol. Learn. Mem..

[16] Bunzeck N, Düzel E (2006). Absolute coding of stimulus novelty in the human substantia nigra/VTA. Neuron.

[17] Bunzeck N, Thiel C (2016). Neurochemical modulation of repetition suppression and novelty signals in the human brain. Cortex.

[18] Bunzeck N, Doeller CF, Dolan RJ, Duzel E (2012). Contextual interaction between novelty and reward processing within the mesolimbic system. Hum. Brain Mapp..

[19] Krebs RM, Heipertz D, Schuetze H, Duzel E (2011). Novelty increases the mesolimbic functional connectivity of the substantia nigra/ventral tegmental area (SN/VTA) during reward anticipation: evidence from high-resolution fMRI. NeuroImage.

[20] Rutishauser U, Mamelak AN, Schuman EM (2006). Single-trial learning of novel stimuli by individual neurons of the human hippocampus-amygdala complex. Neuron.

[21] Kafkas A, Montaldi D (2014). Two separate, but interacting, neural systems for familiarity and novelty detection: a dual-route mechanism. Hippocampus.

[22] Lau JKL, Ozono H, Kuratomi K, Komiya A, Murayama K (2020). Shared striatal activity in decisions to satisfy curiosity and hunger at the risk of electric shocks. Nat. Hum. Behav..

[23] Bromberg-Martin ES, Hikosaka O (2009). Midbrain dopamine neurons signal preference for advance information about upcoming rewards. Neuron.

[24] Ernst D, Becker S, Horstmann G (2020). Novelty competes with saliency for attention. Vision Res..

[25] Eshel N (2015). Arithmetic and local circuitry underlying dopamine prediction errors. Nature.

[26] Schultz W, Dayan P, Montague PR (1997). A neural substrate of prediction and reward. Science.

[27] Lisman JE, Grace AA (2005). The hippocampal-VTA loop: controlling the entry of information into long-term memory. Neuron.

[28] Duszkiewicz AJ, McNamara CG, Takeuchi T, Genzel L (2019). Novelty and dopaminergic modulation of memory persistence: a tale of two systems. Trends Neurosci..

[29] Gruber M. J., Gelman B. D., Ranganath C (2014). States of curiosity modulate hippocampus-dependent learning via the dopaminergic circuit. Neuron.

[30] Takeuchi H (2010). Regional gray matter volume of dopaminergic system associate with creativity: evidence from voxel-based morphometry. NeuroImage.

[31] Lhommée E (2014). Dopamine and the biology of creativity: lessons from Parkinson’s disease. Front. Neurol..

[32] Kulisevsky J, Pagonabarraga J, Martinez-Corral M (2009). Changes in artistic style and behaviour in Parkinson’s disease: dopamine and creativity. J. Neurol..

[33] Inzelberg R (2013). The awakening of artistic creativity and Parkinson’s disease. Behav. Neurosci..

[34] Khalil R, Godde B, Karim AA (2019). The link between creativity, cognition, and creative drives and underlying neural mechanisms. Front. Neural Circuits.

[35] Martinelli C, Rigoli F, Averbeck B, Shergill SS (2018). The value of novelty in schizophrenia. Schizophr. Res..

[36] Hauser MJ, Isbrandt D, Roeper J (2017). Disturbances of novel object exploration and recognition in a chronic ketamine mouse model of schizophrenia. Behav. Brain Res..

[37] Belin D, Belin-Rauscent A, Everitt BJ, Dalley JW (2016). In search of predictive endophenotypes in addiction: insights from preclinical research. Genes Brain Behav..

[38] Belin D, Berson N, Balado E, Piazza PV, Deroche-Gamonet V (2011). High-novelty-preference rats are predisposed to compulsive cocaine self-administration. Neuropsychopharmacology.

[39] Schomaker J (2014). Novelty processing and memory formation in Parkinson׳s disease. Neuropsychologia.

[40] Menza MA, Golbe LI, Cody RA, Forman NE (1993). Dopamine-related personality traits in Parkinson’s disease. Neurology.

[41] Marin RS, Firinciogullari S, Biedrzycki RC (1993). The sources of convergence between measures of apathy and depression. J. Affect. Disord..

[42] Bariselli S (2018). Role of VTA dopamine neurons and neuroligin 3 in sociability traits related to nonfamiliar conspecific interaction. Nat. Commun..

[43] Gunaydin L. A. (2014). Natural neural projection dynamics underlying social behavior. Cell.

[44] Schiemann J (2012). K-ATP channels in dopamine substantia nigra neurons control bursting and novelty-induced exploration. Nat. Neurosci..

[45] Nieh EH (2016). Inhibitory input from the lateral hypothalamus to the ventral tegmental area disinhibits dopamine neurons and promotes behavioral activation. Neuron.

[46] Reynolds GD (2015). Infant visual attention and object recognition. Behav. Brain Res..

[47] Sakaki M, Yagi A, Murayama K (2018). Curiosity in old age: a possible key to achieving adaptive aging. Neurosci. Biobehav. Rev..

[48] Chu L, Tsai JL, Fung HH (2021). Association between age and intellectual curiosity: the mediating roles of future time perspective and importance of curiosity. Eur. J. Ageing.

[49] Dellenbach M, Zimprich D (2008). Typical intellectual engagement and cognition in old age. Neuropsychol. Dev. Cogn. B Aging Neuropsychol. Cogn..

[50] Zimprich D, Allemand M, Dellenbach M (2009). Openness to experience, fluid intelligence, and crystallized intelligence in middle-aged and old adults. J. Res. Pers..

[51] McCrae RR (1999). Age differences in personality across the adult life span: parallels in five cultures. Dev. Psychol..

[52] Ziegler M, Cengia A, Mussel P, Gerstorf D (2015). Openness as a buffer against cognitive decline: the Openness-Fluid-Crystallized-Intelligence (OFCI) model applied to late adulthood. Psychol. Aging.

[53] Donnellan MB, Lucas RE (2008). Age differences in the Big Five across the life span: evidence from two national samples. Psychol. Aging.

[54] Fung HH, Ng SK (2006). Age differences in the sixth personality factor: age differences in interpersonal relatedness among Canadians and Hong Kong Chinese. Psychol. Aging.

[55] Labouvie-Vief G, Diehl M, Tarnowski A, Shen J (2000). Age differences in adult personality: findings from the United States and China. J. Gerontol. B Psychol. Sci. Soc. Sci..

[56] Czigler I, Pató L, Poszet E, Balázs L (2006). Age and novelty: event-related potentials to visual stimuli within an auditory oddball—visual detection task. Int. J. Psychophysiol..

[57] Brodaty H, Altendorf A, Withall A, Sachdev P (2010). Do people become more apathetic as they grow older? A longitudinal study in healthy individuals. Int. Psychogeriatr..

[58] Beard JR (2016). The World report on ageing and health: a policy framework for healthy ageing. Lancet.

[59] Chapman B, Duberstein P, Lyness JM (2007). Personality traits, education, and health-related quality of life among older adult primary care patients. J. Gerontol. B Psychol. Sci. Soc. Sci..

[60] Swan GE, Carmelli D (1996). Curiosity and mortality in aging adults: a 5-year follow-up of the Western Collaborative Group Study. Psychol. Aging.

[61] Ferguson E, Bibby PA (2012). Openness to experience and all-cause mortality: a meta-analysis and *r*_equivalent_ from risk ratios and odds ratios. Br. J. Health Psychol..

[62] Jonassaint CR (2007). Facets of openness predict mortality in patients with cardiac disease. Psychosom. Med..

[63] Turiano NA, Spiro A, Mroczek DK (2012). Openness to experience and mortality in men: analysis of trait and facets. J. Aging Health.

[64] Daffner KR (2006). Increased responsiveness to novelty is associated with successful cognitive aging. J. Cogn. Neurosci..

[65] Menegas W, Akiti K, Amo R, Uchida N, Watabe-Uchida M (2018). Dopamine neurons projecting to the posterior striatum reinforce avoidance of threatening stimuli. Nat. Neurosci..

[66] Molas S (2017). A circuit-based mechanism underlying familiarity signaling and the preference for novelty. Nat. Neurosci..

[67] Bariselli S, Contestabile A, Tzanoulinou S, Musardo S, Bellone C (2018). SHANK3 downregulation in the ventral tegmental area accelerates the extinction of contextual associations induced by juvenile non-familiar conspecific interaction. Front. Mol. Neurosci..

[68] Quirk GJ, Russo GK, Barron JL, Lebron K (2000). The role of ventromedial prefrontal cortex in the recovery of extinguished fear. J. Neurosci..

[69] Sierra-Mercado D, Padilla-Coreano N, Quirk GJ (2011). Dissociable roles of prelimbic and infralimbic cortices, ventral hippocampus, and basolateral amygdala in the expression and extinction of conditioned fear. Neuropsychopharmacology.

[70] Wimmer ME, Hernandez PJ, Blackwell J, Abel T (2012). Aging impairs hippocampus-dependent long-term memory for object location in mice. Neurobiol. Aging.

[71] Shan, Q., Yu, X. & Tian, Y. Reduction of excitatory synaptic transmission efficacy in the infralimbic prefrontal cortex potentially contributes to impairment of contextual fear memory extinction in aged mice. *J. Gerontol. A Biol. Sci. Med. Sci*. **78**, 930–937 (2022). 10.1093/gerona/glac137 35778266

[72] Surmeier DJ, Mercer JN, Chan CS (2005). Autonomous pacemakers in the basal ganglia: who needs excitatory synapses anyway?. Curr. Opin. Neurobiol..

[73] Grace A, Onn S (1989). Morphology and electrophysiological properties of immunocytochemically identified rat dopamine neurons recorded in vitro. J. Neurosci.

[74] Gantz SC, Ford CP, Morikawa H, Williams JT (2018). The evolving understanding of dopamine neurons in the substantia nigra and ventral tegmental area. Annu. Rev. Physiol..

[75] Armbruster BN, Li X, Pausch MH, Herlitze S, Roth BL (2007). Evolving the lock to fit the key to create a family of G protein-coupled receptors potently activated by an inert ligand. Proc. Natl. Acad. Sci. USA.

[76] Urban DJ, Roth BL (2015). DREADDs (designer receptors exclusively activated by designer drugs): chemogenetic tools with therapeutic utility. Annu. Rev. Pharmacol. Toxicol..

[77] Roth BL (2016). DREADDs for neuroscientists. Neuron.

[78] Gomez JL (2017). Chemogenetics revealed: DREADD occupancy and activation via converted clozapine. Science.

[79] Anthony TE (2014). Control of stress-induced persistent anxiety by an extra-amygdala septohypothalamic circuit. Cell.

[80] Morel C (2022). Midbrain projection to the basolateral amygdala encodes anxiety-like but not depression-like behaviors. Nat. Commun..

[81] Matsumoto M, Hikosaka O (2009). Two types of dopamine neuron distinctly convey positive and negative motivational signals. Nature.

[82] Fearnley JM, Lees AJ (1991). Ageing and Parkinson’s disease: substantia nigra regional selectivity. Brain.

[83] Snow BJ (1993). Human positron emission tomographic [18F]fluorodopa studies correlate with dopamine cell counts and levels. Ann. Neurol..

[84] Puopolo M, Raviola E, Bean BP (2007). Roles of subthreshold calcium current and sodium current in spontaneous firing of mouse midbrain dopamine neurons. J. Neurosci..

[85] Branch SY, Sharma R, Beckstead MJ (2014). Aging decreases L-type calcium channel currents and pacemaker firing fidelity in substantia nigra dopamine neurons. J. Neurosci..

[86] Wolfart J, Neuhoff H, Franz O, Roeper J (2001). Differential expression of the small-conductance, calcium-activated potassium channel SK3 is critical for pacemaker control in dopaminergic midbrain neurons. J. Neurosci..

[87] Liss B (2001). Tuning pacemaker frequency of individual dopaminergic neurons by Kv4.3L and KChip3.1 transcription. EMBO J..

[88] Ljungberg T, Apicella P, Schultz W (1992). Responses of monkey dopamine neurons during learning of behavioral reactions. J. Neurophysiol..

[89] Horvitz JC, Stewart T, Jacobs BL (1997). Burst activity of ventral tegmental dopamine neurons is elicited by sensory stimuli in the awake cat. Brain Res..

[90] McNamara CG, Tejero-Cantero Á, Trouche S, Campo-Urriza N, Dupret D (2014). Dopaminergic neurons promote hippocampal reactivation and spatial memory persistence. Nat. Neurosci..

[91] Takeuchi T (2016). Locus coeruleus and dopaminergic consolidation of everyday memory. Nature.

[92] Morrens J, Aydin Ç, van Rensburg AJ, Rabell JE, Haesler S (2020). Cue-evoked dopamine promotes conditioned responding during learning. Neuron.

[93] Rebec GV, Grabner CP, Johnson M, Pierce RC, Bardo MT (1996). Transient increases in catecholaminergic activity in medial prefrontal cortex and nucleus accumbens shell during novelty. Neuroscience.

[94] Legault M, Wise RA (2001). Novelty-evoked elevations of nucleus accumbens dopamine: dependence on impulse flow from the ventral subiculum and glutamatergic neurotransmission in the ventral tegmental area. Eur. J. Neurosci..

[95] Robert P (2009). Proposed diagnostic criteria for apathy in Alzheimer’s disease and other neuropsychiatric disorders. Eur. Psychiatry.

[96] Bastin C, Delhaye E, Moulin C, Barbeau EJ (2019). Novelty processing and memory impairment in Alzheimer’s disease: a review. Neurosci. Biobehav. Rev..

[97] Daffner KR, Scinto LF, Weintraub S, Guinessey JE, Mesulam MM (1992). Diminished curiosity in patients with probable Alzheimer’s disease as measured by exploratory eye movements. Neurology.

[98] Chau SA (2017). Visual selective attention toward novel stimuli predicts cognitive decline in Alzheimer’s disease patients. J. Alzheimer’s Dis..

[99] Lanctôt KL (2017). Apathy associated with neurocognitive disorders: Recent progress and future directions. Alzheimers Dement..

[100] Robert PH (2008). Importance of lack of interest in patients with mild cognitive impairment. Am. J. Geriatr. Psychiatry.

[101] Starkstein SE, Jorge R, Mizrahi R, Robinson RG (2006). A prospective longitudinal study of apathy in Alzheimer’s disease. J. Neurol. Neurosurg. Psychiatry.

[102] Daffner KR (2006). Age-related differences in attention to novelty among cognitively high performing adults. Biol. Psychol..

[103] Daffner KR (2007). Cognitive status impacts age-related changes in attention to novel and target events in normal adults. Neuropsychology.

[104] Fritsch T, Smyth KA, Debanne SM, Petot GJ, Friedland RP (2005). Participation in novelty-seeking leisure activities and Alzheimer’s disease. J. Geriatr. Psychiatry Neurol..

[105] Brookmeyer R, Gray S, Kawas C (1998). Projections of Alzheimer’s disease in the United States and the public health impact of delaying disease onset. Am. J. Public Health.

[106] Hebert LE, Weuve J, Scherr PA, Evans DA (2013). Alzheimer disease in the United States (2010–2050) estimated using the 2010 census. Neurology.

[107] Ambrée O (2009). Levodopa ameliorates learning and memory deficits in a murine model of Alzheimer’s disease. Neurobiol. Aging.

[108] Guzmán-Ramos K (2012). Restoration of dopamine release deficits during object recognition memory acquisition attenuates cognitive impairment in a triple transgenic mice model of Alzheimer’s disease. Learn. Mem..

[109] Shan Q, Hu Y, Chen S, Tian Y (2022). Nucleus accumbens dichotomically controls social dominance in male mice. Neuropsychopharmacology.

[110] Paxinos, G. & Franklin, K. B. J. *The Mouse Brain in Stereotaxic Coordinates,* Version 2 (Academic Press, 2001).

[111] Watson DJG (2012). Selective blockade of dopamine D3 receptors enhances while D2 receptor antagonism impairs social novelty discrimination and novel object recognition in rats: a key role for the prefrontal cortex. Neuropsychopharmacology.

