# Supplementary material for: Reduction in the activity of VTA/SNc dopaminergic neurons underlies aging-related decline in novelty seeking
Source: Commun Biol. 2023 Dec 2;6:1224. doi: 10.1038/s42003-023-05571-x (PMC10693597; doi:10.1038/s42003-023-05571-x)
Supplement: Supplementary file 1 — Supplementary Information [file 42003_2023_5571_MOESM1_ESM.pdf]

Reduction in the activity of VTA/SNc dopaminergic neurons underlies aging-related decline in novelty seeking

Authors
Qiang Shan[1*], Ye Tian[1], Hang Chen[1], Xiaoli Lin[1] and Yao Tian[2]

Affiliation
[1]Laboratory for Synaptic Plasticity, Shantou University Medical College, Shantou, Guangdong, 515041, China
[2]Chern Institute of Mathematics, Nankai University, Tianjin, 300071, China

Correspondence
*Qiang Shan, Laboratory for Synaptic Plasticity, Shantou University Medical College, Shantou, Guangdong, 515041, China; Email: qshan@yahoo.com

Supplementary Information

Supplementary Discussion

The advantage of using the single-object, long-session method with minute-by-minute analysis for investigating novelty-seeking behaviors

Traditionally, most novelty-seeking behavior protocols (in both social and inanimate contexts) reported in the literature employ two different objects (presented sequentially in time, with the one presented in both earlier and later sessions serving as a familiar stimulus in the later session, and the other presented only in the later session serving as a novel stimulus in the later session) that are placed at different locations of a testing chamber in a between-subject design. However, in our experience, mice often show varied inherent basal preference to one object or location over the other, which would bias results. To avoid both issues, we tested novelty-seeking behaviors by employing a single object within one long session. In this within-subject design, a test mouse is exposed to a single object for 20 min. The single object serves as a novel stimulus during the early phase of the session (exposing the test mouse to the object for 2 to 3 minutes is generally sufficient for the mouse to become familiar with the object [1]), and as a familiar stimulus during the late phase of the session (i.e., within-subject comparison).

Most protocols reported in the literature incorporate multiple sessions separated by a period of time that ranges from several minutes to 24 hours (e.g., the protocols reported in Molas et al.[2] and Bariselli et al.[3]). These protocols assume that mice (or other test animals) are able to retain the memory (either working memory or long-term 24-hour memory) of the objects that they encounter in a session into the following session. However, this assumption is possibly untrue for aged mice, which often exhibit impairment in working memory or long-term 24-hour memory [4, 5]. Therefore, using a long-session with the continuous presence of an object is more suitable in the case reported in this study to determine novelty-seeking behaviors of aged mice relative to young mice.

The exploration rate was analyzed minute by minute in this study. Compared to the session-by-session analysis, minute-by-minute analysis can help to separate novelty-driven approach behaviors from neophobia-driven avoidance behaviors, which has clearly been seen in the case of the inanimate novelty in this study (Fig. 1G, 3I, 5I). This practice is especially essential for

aged mice, as their neophobia-driven avoidance behaviors, i.e., the abrupt temporary reduction in the exploration rate, can last for several minutes (starting from the second minute). In such a case, session-by-session (e.g., using 5-minute sessions) analysis would be less likely to reveal differences in novelty-seeking behaviors between conditions (e.g., in Fig. 1G, 3I). Furthermore, the minute-by-minute analysis also allows for determination of whether a condition affects novelty-driven approach behaviors or neophobia-driven avoidance behaviors (e.g., in Fig. 5I). In addition, minute-by-minute analysis can also help to distinguish inanimate novelty seeking behaviors from its blank control, between which the average performance across the whole session can be similar, but the minute-by-minute time course might be different (e.g., in Fig. 5I versus Fig. S3).

Supplementary References

1. Zhang, K., Bromberg-Martin, E. S., Sogukpinar, F., Kocher, K. & Monosov, I. E. Surprise and recency in novelty detection in the primate brain. *Curr. Biol.* **32**, 2160–2173.e6 (2022).
2. Molas, S. et al. A circuit-based mechanism underlying familiarity signaling and the preference for novelty. *Nat. Neurosci.* **20**, 1260–1268 (2017).
3. Bariselli, S. et al. Role of VTA dopamine neurons and neuroligin 3 in sociability traits related to nonfamiliar conspecific interaction. *Nat. Commun.* **9**, 3173 (2018).
4. Grady, C. The cognitive neuroscience of ageing. *Nat. Rev. Neurosci.* **13**, 491–505 (2012).
5. Morrison, J. H. & Baxter, M. G. The ageing cortical synapse: hallmarks and implications for cognitive decline. *Nat. Rev. Neurosci.* **13**, 240–250 (2012).

Supplementary Figures

Fig. S1 (supplementary to Fig. 1)

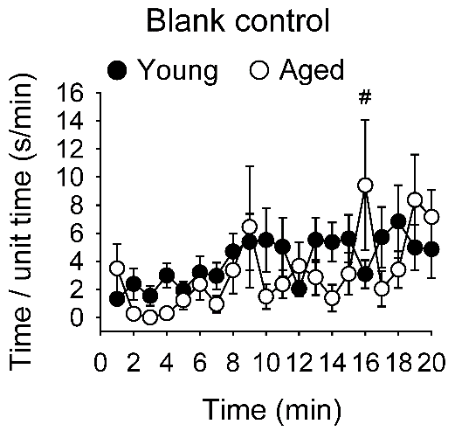

Fig. S1 (supplementary to Fig. 1). The courses of time young and aged mice spent per unit time in the central area of the test chamber

The data were analyzed using two-way repeated measures ANOVA: $F_{1,14} = 0.53$, $p = 0.48$ for age effect; $F_{19,266} = 2.4$, $p < 0.001$ for time effect; $F_{19,266} = 1.6$, $p = 0.059$ for age × time interaction; post-hoc test, $^{\#}p < 0.05$; n = 8 each. All data are presented as mean ± SEM.

Fig. S2 (supplementary to Fig. 3)

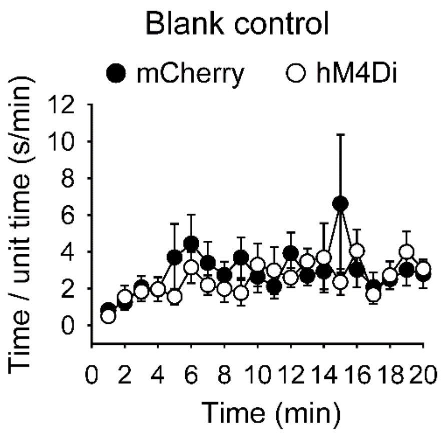

Fig. S2 (supplementary to Fig. 3). The courses of time hM4Di-mCherry- and mCherry-expressing young DAT-Cre mice spent per unit time in the central area of the test chamber

The data were analyzed using two-way repeated measures ANOVA: $F_{1,16} = 0.50$, $p = 0.49$ for chemogenetic effect; $F_{19,304} = 1.5$, $p = 0.091$ for time effect; $F_{19,304} = 0.85$, $p = 0.64$ for chemogenetic × time interaction; n = 9 each. All data are presented as mean ± SEM.

Fig. S3 (supplementary to Fig. 5)

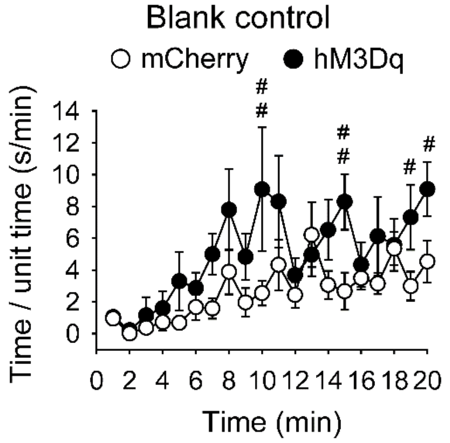

Fig. S3 (supplementary to Fig. 5). The courses of time hM3Dq-mCherry- and mCherry-expressing aged DAT-Cre mice spent per unit time in the central area of the test chamber

The data were analyzed using two-way repeated measures ANOVA: $F_{1,18} = 5.3$, $p = 0.033$ for chemogenetic effect; $F_{19,342} = 4.9$, $p < 0.001$ for time effect; $F_{19,342} = 1.2$, $p = 0.22$ for chemogenetic × time interaction; post-hoc test, $^{##}p < 0.01$, $^{#}p < 0.05$; n = 10 each. All data are presented as mean ± SEM.
