# Supplementary material for: Reduction in the activity of VTA/SNc dopaminergic neurons underlies aging-related decline in novelty seeking
Source: Commun Biol. 2023 Dec 2;6:1224. doi: 10.1038/s42003-023-05571-x (PMC10693597; doi:10.1038/s42003-023-05571-x)
Supplement: Supplementary file 2 — Description of Supplementary Materials [file 42003_2023_5571_MOESM2_ESM.docx]

**Description of Additional Supplementary Files**

**File name:** Supplementary Data

**Description:** The source data underlying the graphs in the paper
